# Constriction of the mitochondrial inner compartment is a priming event for mitochondrial division

**DOI:** 10.1038/ncomms15754

**Published:** 2017-06-09

**Authors:** Bongki Cho, Hyo Min Cho, Youhwa Jo, Hee Dae Kim, Myungjae Song, Cheil Moon, Hyongbum Kim, Kyungjin Kim, Hiromi Sesaki, Im Joo Rhyu, Hyun Kim, Woong Sun

**Affiliations:** 1 Department of Anatomy, Korea University College of Medicine, 145 Anam-ro, Seongbuk-gu, Seoul 02841, Republic of Korea; 2 Department of Brain & Cognitive Sciences, Daegu Gyeongbuk Institute of Science and Technology, 333 Techno Jungang-daero, Hyeonpung-myeon, Dalseong-gun, Daegu 42988, Republic of Korea; 3 Department of Biological Sciences, Seoul National University, Seoul 08826, Republic of Korea; 4 Department of Pharmacology, Yonsei University College of Medicine, 50-1 Yonsei-ro, Seodaemoon-gu, Seoul 03722, Republic of Korea; 5 Korea Brain Research Institute, 61 Choeomdan-Ro, Dong-Gu, Daegu 41068, Republic of Korea; 6 Department of Cell Biology, Johns Hopkins University School of Medicine, Baltimore, Maryland 21205, USA

## Abstract

Mitochondrial division is critical for the maintenance and regulation of mitochondrial function, quality and distribution. This process is controlled by cytosolic actin-based constriction machinery and dynamin-related protein 1 (Drp1) on mitochondrial outer membrane (OMM). Although mitochondrial physiology, including oxidative phosphorylation, is also important for efficient mitochondrial division, morphological alterations of the mitochondrial inner-membrane (IMM) have not been clearly elucidated. Here we report spontaneous and repetitive constriction of mitochondrial inner compartment (CoMIC) associated with subsequent division in neurons. Although CoMIC is potentiated by inhibition of Drp1 and occurs at the potential division spots contacting the endoplasmic reticulum, it appears on IMM independently of OMM. Intra-mitochondrial influx of Ca^2+^ induces and potentiates CoMIC, and leads to K^+^-mediated mitochondrial bulging and depolarization. Synergistically, optic atrophy 1 (Opa1) also regulates CoMIC via controlling Mic60-mediated OMM–IMM tethering. Therefore, we propose that CoMIC is a priming event for efficient mitochondrial division.

## Abstract (web summary)

The role of morphological alterations in the mitochondrial inner-membrane in regulating mitochondrial division are unknown. Here, the authors describe spontaneous and repetitive constriction of the mitochondrial inner compartment, and suggest this acts as a priming event for efficient mitochondrial division.

Mitochondria continuously undergo fusion and division to regulate proper function, quality and distribution in response to changing cellular environments^1^. According to the endosymbiosis theory, the outer membrane (OMM) and inner membrane (IMM) of mitochondria have different chemical and molecular properties^2^. Thus, regulation of mitochondrial morphology requires the coordination of distinct machineries for OMM and IMM^3^. In mammalian cells, mitochondrial fusion is mediated by two sequential steps: OMM fusion by mitofusin 1/2 (Mfn1/2), and IMM fusion by optic atrophy 1 (Opa1). In contrast, mitochondrial division is known to be solely driven by dynamin-related protein 1 (Drp1). Drp1 translocates from the cytosol to OMM receptors including Fis1, Mff and MiD49/51 (ref. 4), and forms spiral-like structure by oligomerization^5^. Subsequently, this spiral structure constricts and divides a mitochondrion. However, structural biological studies have revealed that the Drp1 spiral is not sufficient for the initiation of mitochondrial constriction because mitochondrial diameter (0.5–1.0 μm) is much thicker than the Drp1 spiral (∼100 nm)^5^,^6^, implying the requirement for an initial mitochondrial constriction step before Drp1 action. Recent reports have revealed that actin filaments promote initial mitochondrial constriction through direct contact of mitochondria with the endoplasmic reticulum (ER)^7^,^8^, and dynamin 2 collaborates with Drp1 for finalization of mitochondrial division^9^. This mode of action is similar to endocytosis mediated by dynamin^10^, and recent reports have demonstrated that Drp1 is involved in endocytosis as well as mitochondrial division^11^,^12^. These findings suggest that Drp1-dependent mitochondrial division evolutionarily originated from cytosolic vesicle-scissoring machineries^2^.

Endosymbiosis theory proposes that the IMM is evolutionarily derived from a prokaryote-like endosymbiont, while the OMM is derived from the plasma membrane^2^. Prokaryotic division is executed by FtsZ, a tubulin-like protein, forming intracellular Z-ring^2^. Consistently, primitive eukaryotes, such as red algae, have mitochondrial orthologues of FtsZ, and they exhibit a Z-ring during IMM constriction in the mitochondrial matrix before Drp1-mediated ring formation^13^. This information implies that mitochondria require another step in constriction of the IMM before the activation of cytosolic Drp1. However, neither FtsZ-like proteins nor Z-ring structures have been identified in mammals^2^. Nonetheless, constriction or division of the IMM in the absence of Drp1 has been observed in yeast^14^, *C. elegans*^15^ and mouse fibroblasts^16^,^17^. Also, electron microscopy studies have shown that the IMM can divide in the absence of OMM division^18^. Although these reports support the notion that OMM-independent intra-mitochondrial event(s) may exist, its presence in mammalian cells and the underlying mechanisms have not been elucidated.

Numerous light-scattering experiments using isolated mitochondria have shown that intra-mitochondrial homoeostasis of cations, such as Ca^2+^, K^+^ and Na^+^, is important for the regulation of mitochondrial volume, causing changes in mitochondrial function^19^. In particular, K^+^ influx into the mitochondria induces swelling or bulging via osmosis. In addition, mitochondrial dysfunction by mitochondrial depolarization and Ca^2+^ overload induces Drp1-dependent mitochondrial fragmentation, which contributes to mitophagy or apoptosis^20^. In contrast, metabolically active mitochondria are elongated and are not readily fragmented by intracellular stressors, which is the result of enhanced fusion with Mfn1/2 and Opa1 and decreased activity of Drp1 (refs 21, 22). Recent papers have reported that elongated mitochondria transiently form bead-on-a-string shapes with fluctuations in intra-mitochondrial metabolic status, membrane potential, ROS production and Ca^2+^ influx^22^,^23^,^24^,^25^. However, the significance of these morphological changes and their possible association with mitochondrial division has not yet been addressed.

In this study, we report that the transient changes in mitochondrial morphology to bead-on-a-string shape are primarily caused by intra-mitochondrial morphological alterations, referred to as Constriction of Mitochondrial Inner Compartments (CoMIC). While this intra-mitochondrial event occurs at ER–mitochondrial contact sites, it appears to be initiated independently of Drp1 action and OMM constriction. We provide evidence that CoMIC is initiated by an influx of Ca^2+^ into the mitochondria, which mediates subsequent mitochondrial influx of K^+^ and Opa1 activation of the IMM. We propose that this regulatory mechanism can coordinate IMM morphological changes with OMM constriction for efficient mitochondrial division.

## Results

### Elongated mitochondria exhibit CoMIC before division

**Figure 1:**
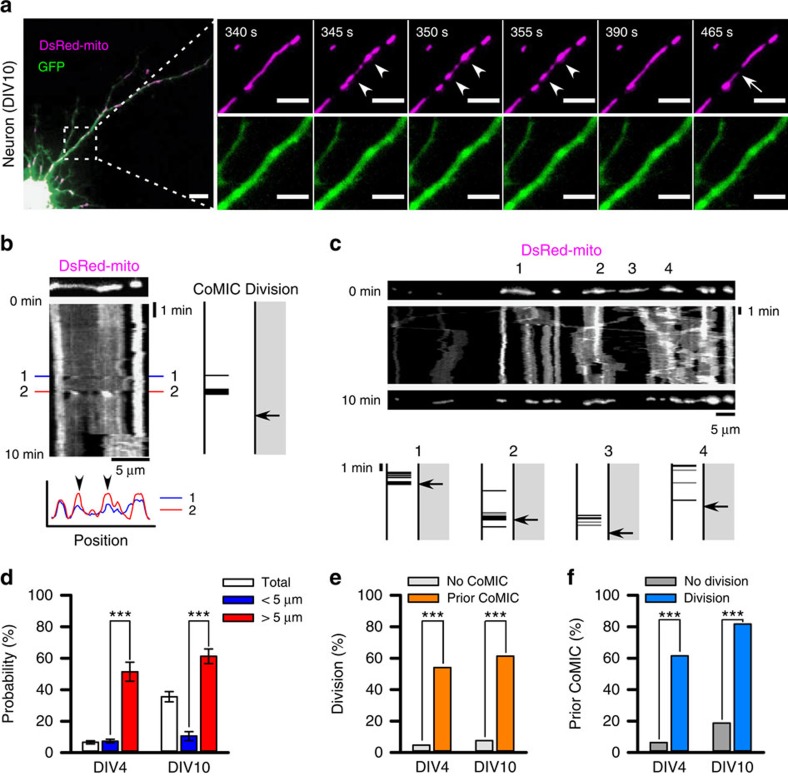
CoMIC appears in elongated mitochondria before mitochondrial division. (**a**) Time-lapse images of mitochondrial matrices (DsRed-mito) in neuronal process (GFP). The white arrowheads and the arrow indicate sites of mitochondrial bulging and division, respectively. Scale bars, 10 and 5 μm in the lower and higher magnification images, respectively. (**b**) Kymograph of DsRed-mito in **a**. The lower graph is the intensity profile at two CoMIC-showing time points (1, 2), and arrow heads indicate conserved bulging site. Two illustrations on the right indicate the pattern of CoMIC and occurrence of division. CoMIC pattern and division are labelled by spikes and an arrow, respectively. (**c**) Kymograph of DsRed-mito in a neuronal process. The upper numbers indicate mitochondria showing CoMIC. The lower panels show the pattern of CoMIC and occurrence of division in mitochondria labelled with the numbers above. (**d**) Probability of CoMIC in mitochondria from young (DIV4; <5 μm=530 mitochondria, >5 μm=70 mitochondria) and mature neurons (DIV10; <5 μm=114 mitochondria, >5 μm=111 mitochondria) during a 10-min time period (***P<0.01 by the Mann–Whitney rank sum test). Error bars indicate s.e. (**e**) Proportion of mitochondria undergoing subsequent division among mitochondria showing no or prior CoMIC (***P<0.01 by the *χ*^2^-test with Yates's correction for continuity). (**f**) Proportion of mitochondria showing prior CoMIC among non-dividing or dividing mitochondria (***P<0.01 by *χ*^2^-test with Yates's correction for continuity).

To explore the intra-mitochondrial events that take place during mitochondrial division, we performed time-lapse imaging in rat cultured cortical neurons expressing DsRed-mito, which labels the mitochondrial matrix^26^. Interestingly, a subset of long mitochondria in neuronal processes spontaneously exhibited repetitive and reversible beads-on-a-string-like structures accompanying focal constriction and bulging (Fig. 1a and Supplementary Movie 1). On the other hand, co-expressed green fluorescent protein (GFP) signals were not altered, ruling out the possibility that the observed mitochondrial change is influenced by morphological alteration of neuronal processes. This phenomenon was also observed in other cell types, including 293T, HeLa and HepG2 cells (Supplementary Fig. 1a), indicating that CoMIC is common to diverse cell types and species. Through kymographic analysis, we temporally and spatially analyzed the occurrence of CoMICs and mitochondrial division. The bulged and constricted sites were spatially conserved during repetitive CoMICs (Fig. 1b), and each mitochondrion exhibited unique and unsynchronized patterns of CoMIC spikes (Fig. 1c). These properties imply that CoMICs may be an intrinsic property of mitochondria. In young neurons (days *in vitro* 4, DIV4), the probability that a mitochondrion shows a CoMIC in a 10-min time period was 12.3%, and in mature neurons (DIV10), which have more elongated mitochondria^27^, the probability was 35.6% (Fig. 1d). Notably, mitochondria longer than 5 μm have a significantly higher probability of CoMIC (young neurons, 50%; mature neurons, 61.3%) than do shorter mitochondria (young neurons, 7.4%; mature neurons, 10.5%) (Fig. 1e). This indicates that elongated mitochondria are prone to CoMIC. In addition, mitochondria exhibiting CoMIC often underwent subsequent division at only one of multiple constricted sites (Fig. 1a–c). Further quantitative analyses revealed that 54.1 and 61.3% of mitochondria exhibiting CoMIC underwent subsequent division in young and mature neurons, respectively (Fig. 1e), and that larger proportions (61.5% and 81.7%, respectively) of dividing mitochondria exhibited prior CoMIC (Fig. 1f). These results suggest that CoMIC is closely correlated with mitochondrial division.

### CoMIC is potentiated by suppression of Drp1

**Figure 2:**
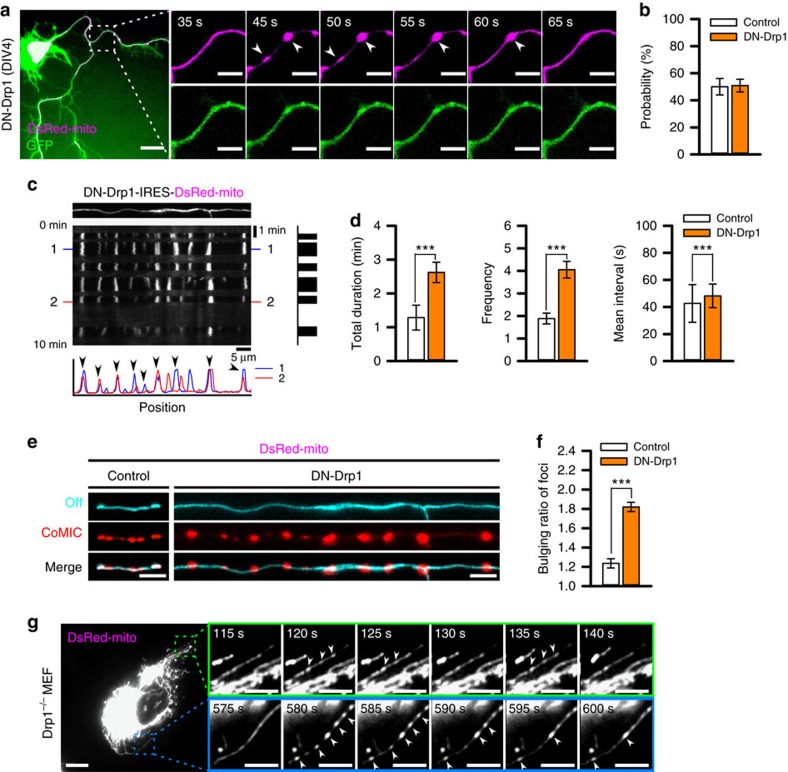
CoMIC is potentiated by inhibition of Drp1, but does not depend on Drp1. (**a**) Time-lapse images of the mitochondrial matrix and neuronal processes in DN-Drp1-expressing neurons (DIV4). Arrowheads indicate sites of mitochondrial bulging. Scale bars, 10 and 5 μm in the lower and higher magnification images, respectively. (**b**) Probability of CoMIC in elongated mitochondria (>5 μm) as compared with control (70 mitochondria) and DN-Drp1-expressing neurons (114 mitochondria). Error bar indicate s.e. (**c**) Representative kymographic analysis of a mitochondrion showing CoMIC in a DN-Drp1-expressing neuron. Arrow heads indicate conserved bulging site on two CoMIC-showing time points (1, 2). (**d**) Quantification of the total duration, frequency and mean interval of CoMIC in control and DN-Drp1-expressing neurons (***P<0.01 by Mann–Whitney rank sum test). (**e**) Straightened images of DsRed-mito showing minimal bulging (cyan signal) and maximal bulging (red signal) in control and DN-Drp1-expressing neurons. (**f**) Bulging ratio in foci during CoMIC in control and DN-Drp1-expressing neurons (***P<0.01 by Mann–Whitney rank sum test). (**g**) CoMIC in Drp1 knockout mouse embryonic fibroblasts. The time-lapse panels are enlarged in the green and blue boxes. Arrowheads indicate the sites of mitochondrial bulging. Scale bars, 10 and 5 μm in the lower and higher magnification images, respectively.

We further examined the association of CoMIC with mitochondrial division by overexpression of a GTPase-defective mutant of Drp1 (DN-Drp1), which inhibits the activity of endogenous Drp1 (ref. 28), resulting in marked elongation of mitochondria with a reduced rate of division (Supplementary Fig. 2). By using other chemical and genetic dyes, we confirmed that the CoMIC was real but not DsRed-specific artifact (Supplementary Fig. 3). Strikingly, DN-Drp1-expressing neurons exhibited potentiated frequency and duration of CoMIC compared with control neurons (Fig. 2a and Supplementary Movie 2), although the probability of CoMIC was not affected when compared with elongated mitochondria (Fig. 2b). This phenomenon was also found in other cell types, including 293T, HeLa and HepG2 cells (Supplementary Fig. 1b), implying that the CoMIC-enhancing effect of DN-Drp1 is conserved in cells of diverse types and species. Kymographic analysis revealed that CoMIC was more frequent and prolonged in the mutant compared with control neurons (Fig. 2c). Further quantitative analyses showed significant increases in total duration, frequency and mean interval of CoMIC in DN-Drp1-expressing neurons compared with control neurons (Fig. 2d). In addition, mitochondrial bulging during CoMIC was also enhanced by DN-Drp1 (Fig. 2e,f). These results show that Drp1 relieves mitochondria from the tension caused by CoMIC by executing mitochondrial division. It is known that DN-Drp1 maintains strong oligomerization activity and affinity for the lipid membrane^29^. In addition, photo-damage or hypoxic stress may induce CoMIC-like morphology resulting from inhibition of Drp1 on the OMM^22^,^25^. Therefore, we wondered if the overexpression of DN-Drp1 promoted CoMIC via enhanced mitochondrial constriction with perturbation of division. However, knockdown of endogenous Drp1 with a small hairpin RNA (shRNA) similarly promoted CoMIC (Supplementary Fig. 4). Crucially, Drp1-knockout mouse embryonic fibroblasts also exhibited CoMIC (Fig. 2g). These data suggest that Drp1 is not required for the initiation of CoMIC, but may terminate CoMIC cycles by mitochondrial division.

### ER-mitochondria contact site spatially specifies CoMIC spots

**Figure 3:**
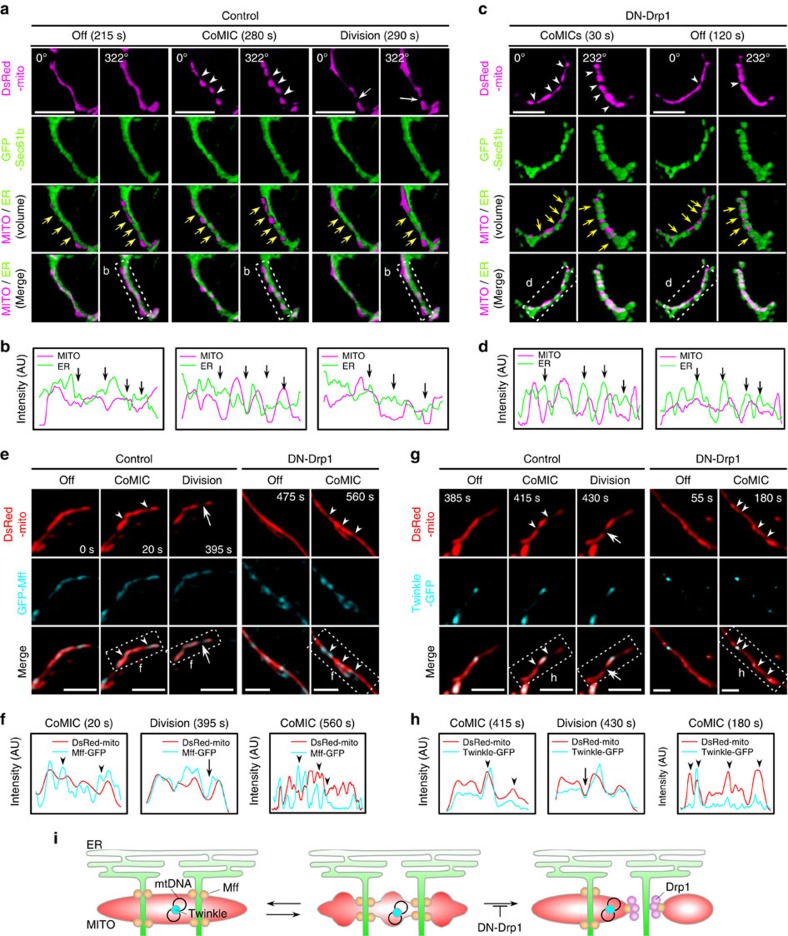
CoMIC is spatially associated with the ER. (**a**,**c**) 3D images of mitochondria and ER (GFP-Sec61b) during off-phase, CoMIC and mitochondrial division in control (**a**) and DN-Drp1-expressing neurons (**c**). Scale bar, 5 μm. White arrowheads and arrows indicate the spots of CoMIC and division, respectively. Yellow arrows indicate the spots that are enclosed by the ER. (**b**,**d**) Intensity profiles of the mitochondrial matrix and the ER in the dotted boxes in **a**,**c**, respectively. (**e**,**g**) Representative images of mitochondria, GFP-Mff (**e**), Twinkle–GFP (**g**) during off-phase, CoMIC and mitochondrial division in control and DN-Drp1-expressing neurons. Scale bar, 5 μm, and white arrowheads and arrows indicate the spots of CoMIC and division, respectively. (**f**,**h**) Intensity profiles of the mitochondrial matrix, GFP-Mff and Twinkle–GFP in the dotted boxes in **e**,**g**, respectively. (**i**) A hypothetical model of mitochondrial association with the ER during CoMIC.

Recent studies revealed that physical association of ER–mitochondria and actin filaments can trigger mitochondrial constriction before recruitment of Drp1 (refs 7, 8). Our kymographic analyses showed that mitochondrial constriction occurs in spatially conserved sites throughout repetitive CoMIC (Figs 1e and 2b), implying the presence of structural foci for CoMIC. We thus postulated that spatial association with the ER may specify the position at which CoMIC occurs. As expected, CoMIC and subsequent mitochondrial division occurred when mitochondria spatially intersected with or were enclosed by the ER, as assessed by labelling with GFP-Sec61b (Fig. 3a,b and Supplementary Fig. 5a). DN-Drp1-expressing neurons and 293T cells also exhibited CoMIC at ER–mitochondrial intersections (Fig. 3c,d, Supplementary Movie 3 and Supplementary Fig. 5b–d). In addition, GFP-Mff, which marks putative sites of constriction and division in ER–mitochondria contact site^7^, localized on constriction sites during CoMIC in control and DN-Drp1-expressing neurons (Fig. 3e,f). More recently, it has been demonstrated that replication of mitochondrial DNA (mtDNA) is spatially associated with mitochondrial division in ER–mitochondria contact site^30^. By using GFP-tagged Twinkle which is mtDNA helicase for replication^31^,^32^, we examined the positions where mtDNA replisomes localize during CoMIC. In off-phase, Twinkle–GFP signals were localized on the elongated mitochondria, and mitochondrial constrictions occurred juxtaposition of Twinkle–GFP during CoMIC (Fig. 3g,h). Therefore, we suggest that structural specification of CoMIC is associated with ER–mitochondrial contact site. Remarkably, however, during CoMIC, the spatial intersections between the ER and mitochondria seems to be maintained regardless of phase, and some intersections did not show any CoMIC (Fig. 3a–d and Supplementary Fig. 5b). However, depletion of Mfn2, which regulates ER–mitochondria contact^33^,^34^, did not modify DN-Drp1-potentiated CoMIC (Supplementary Fig. 6), similar to previous report^7^. These data indicate that contact with the ER *per se* does not induce CoMIC but most likely plays a role in the structural specification of CoMIC (Fig. 3i). We further examined whether actin filaments are involved in induction of CoMIC because actin polymerization triggers constriction of the OMM before Drp1-dependent division^8^. However, treatment with latrunculin B, which disrupts actin filaments, did not modify CoMIC (Supplementary Fig. 7). Collectively, these data suggest that the cytosolic vesicle-forming machinery, such as actin filaments and Drp1, are not involved in the induction of CoMIC, while ER–mitochondrial intersections spatially specify the foci of CoMIC.

### CoMIC is independent on OMM changes

**Figure 4:**
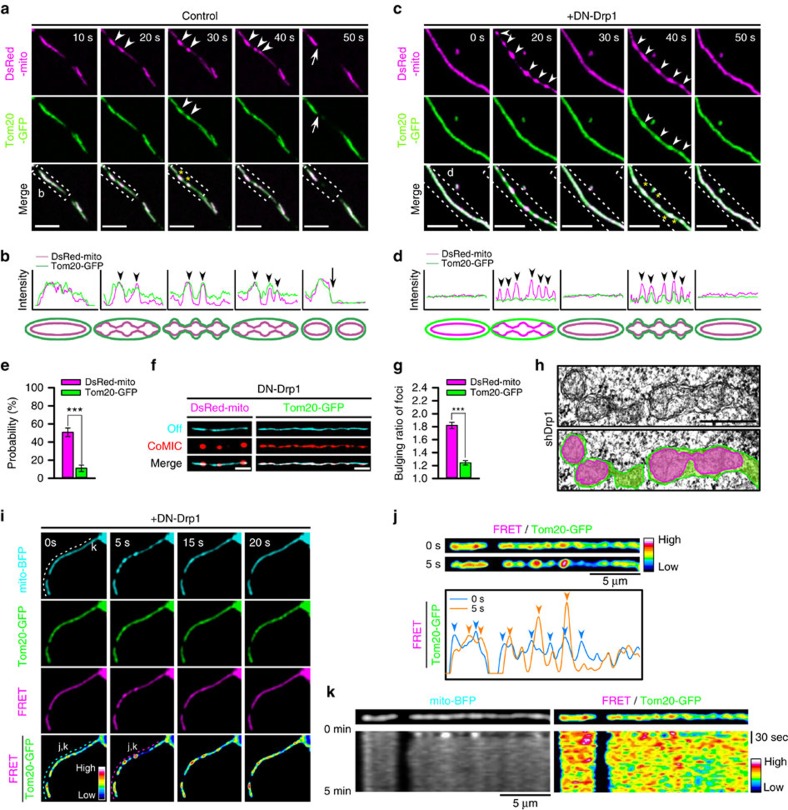
CoMIC is OMM-independent event accompanying rearrangement on IMM–OMM contact. (**a**,**c**) Time-lapse images of the mitochondrial matrix and OMM (Tom20-GFP) in control (**a**) and DN-Drp1-expressing neurons (**c**). Arrowheads and arrows indicate the bulging sites of mitochondria during CoMIC and mitochondrial division, respectively. Yellow asterisks indicate synchronized bulging of the OMM and matrix. Scale bar, 5 μm. (**b**,**d**) Intensity profiles (upper graphs) and schematic diagrams (lower graphs) of the mitochondrial matrix (magenta lines) and OMM (green lines) in the dotted insects shown in **a**,**c**, respectively. (**e**) Probability of CoMIC (114 mitochondria) and OMM constriction (81 mitochondria) in elongated mitochondria from DN-Drp1-expressing neurons (***P<0.01 by Mann–Whitney rank sum test). Error bar indicate s.e. (**f**) Straightened images of DsRed-mito and Tom20-GFP showing minimal (cyan signal) and maximal bulging (red signal) in a DN-Drp1-expressing neuron. (**g**) Bulging ratio in foci of DsRed-mito and Tom20-GFP during CoMIC in DN-Drp1-expressing neurons (***P<0.01 by Mann–Whitney rank sum test). (**h**) Electron microscopy of mitochondria in neuron infected by shDrp1-virus. Scale bar, 500 nm. Green and magenta shades indicate area marked by OMM and IMM, respectively. (**i**) Time-lapse images for FRET-based proximity analysis between OMM and mitochondrial matrix, which are labelled by Tom20-GFP and mito-BFP, in a DN-Drp1-expressing neuron. Scale bar, 5 μm. FRET ratio (FRET/Tom20-GFP) is pseudo-coloured. (**j**) Straightened images of FRET/Tom20-GFP during off-phase and CoMIC of mitochondria, and their intensity profiles (lower panel). Arrows indicate hot spots of FRET ratio. (**k**) Kymographies of mito-BFP and FRET/Tom20-GFP of mitochondria on dotted line of **i**.

Because cytosolic fission machinery is not involved in CoMIC, we wondered the behaviour of the OMM during CoMIC. Interestingly, a subset of OMM, which was labelled with Tom20-GFP, in elongated mitochondria exhibited neither constriction nor bulging during CoMIC in control neurons, but underwent subsequent division (Fig. 4a,b nd Supplementary Fig. 8a). Such dissociation of CoMIC and OMM changes was also observed in 293T cells (Supplementary Fig. 8b). In some cases, CoMIC and OMM constrictions were simultaneously observed, along with conservation of bulging spots. Similarly, neurons and 293T cells expressing DN-Drp1 exhibited no or marginal OMM bulging during potentiated CoMIC (Fig. 4c,d, Supplementary Movie 4 and Supplementary Fig. 8c). The quantification confirmed that the probability of focal OMM constriction was much lower than that of CoMIC (Fig. 4e). In addition, bulging of the OMM is weaker than that of CoMIC (Fig. 4f,g), implying the dissociation of OMM and IMM at constriction sites during CoMIC. Supporting this, electron microscopy in shDrp1-infected neuron showed that some elongated mitochondria exhibit focal separation of IMM and OMM (Fig. 4h). To visualize the distance of OMM–IMM during CoMIC, we performed proximity assay by fluorescence resonance energy transfer (FRET) between mitochondria matrix and OMM, labelled by mito-BFP and Tom20-GFP, respectively (Supplementary Fig. 9). We expected that proximity between IMM and OMM may be represented by FRET signal, and found some hot and cold spots were shown on mitochondria (Fig. 4i). Strikingly, among the hot spots, bulging of mitochondrial matrix occurred at some spots with enhanced proximity between OMM and IMM during CoMIC compared with off-phase (Fig. 4i,j). After CoMIC, these hot spots returned to their basal level. On the other hand, the cold spots became striking at the constriction sites during CoMIC (Fig. 4i,j), indicating that constriction of mitochondrial matrix increases OMM–IMM distances. Notably, the hot and cold spots appeared to be spatiotemporally conserved with moderate dynamicity during off-phase (Fig. 4k). Therefore, these data suggest that CoMIC induces transient disorganization of OMM–IMM contact.

### Mitochondrial Ca^2+^ initiates and potentiates CoMIC

**Figure 5:**
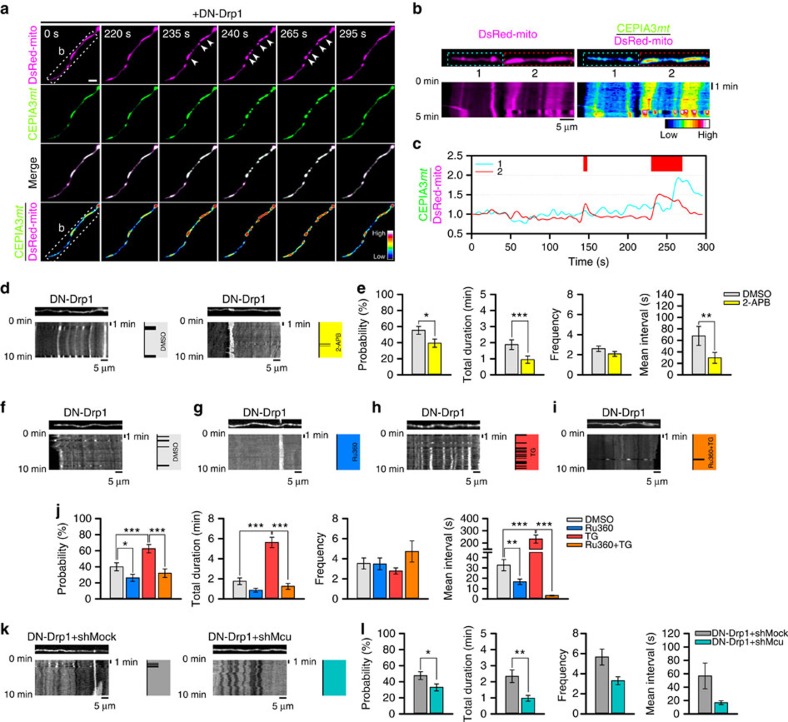
Intra-mitochondrial Ca^2+^ regulates CoMIC. (**a**) Time-lapse images of DsRed-mito and CEPIA3*mt* in DN-Drp1-expressing neuron. Arrow heads indicate mitochondrial bulging. Scale bar, 5 μm, and CEPIA3*mt*/DsRed-mito is pseudo-coloured. (**b**) Kymographies of DsRed-mito and CEPIA3*mt*/DsRed-mito of mitochondria in dotted box of **a**. (**c**) Temporal CEPIA3*mt*/DsRed-mito fluctuation of mitochondria of dotted boxes (#1, #2) of **b**. Upper red boxes indicate CoMIC duration of mitochondrion labelled by #2. (**d**) Representative kymographies of mito-GFP and patterns of CoMIC (right diagram) in DN-Drp1-expressing neurons treated with DMSO and 2-APB (1 μM) for 1 h. (**e**) Probability of CoMIC in elongated mitochondria treated with DMSO (103 mitochondria) and 2-APB (94 mitochondria), and quantification of the total duration, frequency and average single duration of CoMIC (***P<0.01 and *P<0.05 by Mann–Whitney rank sum test). Error bar indicate s.e. (**f**–**i**) Representative kymographies of DsRed-mito and patterns of CoMIC (right diagram) in DN-Drp1-expressing neurons treated with DMSO (**f**), Ru360 (10 μM) (**g**), thapsigargin (TG, 1 μM) (**h**) and TG with 30 min pretreatment with Ru360 (Ru360+TG) (**i**) for 1 h. (**j**) Probability of CoMIC in elongated mitochondria treated with DMSO (100 mitochondria), Ru360 (95 mitochondria), TG (88 mitochondria) or Ru360+TG (95 mitochondria), and quantification of the total duration, frequency and average single duration of CoMIC (***P<0.01, **P<0.02 and *P<0.05 by Mann–Whitney rank sum test). (**k**) Representative kymographies of DsRed-mito and patterns of CoMIC (right diagram) in DN-Drp1+shMock- and DN-Drp1+shMcu-expressing neurons. (**l**) Probability of CoMIC in elongated mitochondria from DN-Drp1+shMock- (105 mitochondria) and DN-Drp1+shMcu-expressing neurons (118 mitochondria), and quantification of the total duration, frequency and average single duration of CoMIC (**P<0.02 and *P<0.05 by Mann–Whitney rank sum test).

Spatial association of CoMIC with ER may indicate the Ca^2+^-mediated functional linkage between CoMIC and ER. Especially, intra-mitochondrial Ca^2+^ entry is implicated in many mitochondrial functions, including respiration, apoptosis, reactive oxygen species production and mitochondrial transport^35^,^36^. To investigate involvement of intra-mitochondrial Ca^2+^ in CoMIC, we monitored the level of intra-mitochondrial Ca^2+^ during CoMIC by using CEPIA3*mt* (ref. 37). Notably, intra-mitochondrial Ca^2+^ was transiently increased just before the CoMIC in DN-Drp1-expressing neuron, and returned to basal level in off-phase (Fig. 5a–c, mitochondrion #2). However, some mitochondria did not undergo CoMIC in spite of mitochondrial Ca^2+^ rise (Fig. 5a–c, mitochondrion #1). It implies that mitochondrial Ca^2+^ signal may be necessary, but not sufficient, for CoMIC. Next, we examined the role of Ca^2+^ in CoMIC. Treatment with BAPTA-AM, a cell-permeable Ca^2+^ chelator, completely inhibited CoMIC in DN-Drp1-expressing neurons (Supplementary Fig. 10a,b). However, ethylene glycol-bis(β-aminoethyl ether)-N,N,N′,N′-tetraacetic acid (EGTA), a non-cell-permeable Ca^2+^ chelator, did not affect CoMIC (Supplementary Fig. 10c). In contrast, A23187, which is a Ca^2+^ ionophore, induced robust CoMIC and eventual mitochondrial fragmentation (Supplementary Fig. 10d). These data indicate that intracellular Ca^2+^ rise can induce CoMIC. In consistent, 2-APB, which is an inhibitor of IP_3_ receptor releasing Ca^2+^ from ER, significantly declined the probability of basal CoMIC and shortened its total and average single durations (Fig. 5d,e), implying the contribution of intra-mitochondrial Ca^2+^ shuttling from ER to CoMIC. To more selectively explore the involvement of intra-mitochondrial Ca^2+^ entry in CoMIC initiation, we treated DN-Drp1-expressing neurons with Ru360, an inhibitor of mitochondrial Ca^2+^ uptake. Remarkably, treatment with Ru360 inhibited CoMIC as compared with vehicle (DMSO) treatment (Fig. 5f,g). Quantitative analyses revealed that Ru360 significantly reduced the probability of CoMIC, and shortened the average duration of an individual CoMIC (Fig. 5j). Furthermore, Ru360 efficiently blocked CoMIC induced by thapsigargin (TG), which increases intracellular Ca^2+^ concentration by blocking calcium uptake via inhibition of sarco/ER Ca^2+^ ATPase (Fig. 5h-j). Similarly, depletion of Mcu by specific shRNA, which is mitochondrial uniporter, decreased the probability of CoMIC and shortened total duration of CoMIC (Fig. 5k,l), although it did not affect mitochondrial morphology (Supplementary Fig. 11). Taken together, these data suggest that intra-mitochondrial Ca^2+^ entry is required for the induction and potentiation of CoMIC.

### Mitochondrial Ca^2+^ promotes efficient mitochondrial division

**Figure 6:**
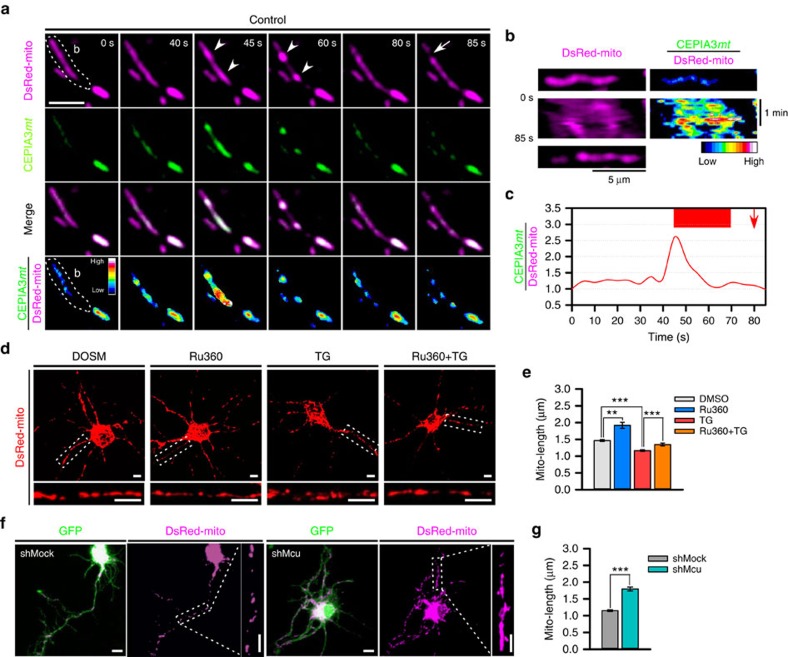
Intra-mitochondrial Ca^2+^ contributes to efficient mitochondrial division. (**a**) Time-lapse images of DsRed-mito and CEPIA3*mt* in control neuron. Scale bar, 5 μm, and CEPIA3*mt*/DsRed-mito is pseudo-coloured. Arrow heads and arrow indicate mitochondrial bulging and division, respectively. (**b**) Kymographies of DsRed-mito and CEPIA3*mt*/DsRed-mito of mitochondria in dotted region of **a**. (**c**) Temporal CEPIA3*mt*/DsRed-mito fluctuation of a mitochondrion of dotted region of **a**. Upper red box and arrow indicate CoMIC duration and mitochondrial division, respectively. (**d**) Representative morphology of mitochondria in neurons treated with DMSO and Ru360 with or without TG. The lower, enlarged panels are straightened images from the boxes in the upper panels. Scale bar, 5 μm. (**e**) Average mitochondrial length following treatment with DMSO (2,415 mitochondria), Ru360 (635 mitochondria), TG (1,438 mitochondria) and TG+Ru360 (706 mitochondria). (***P<0.01 and **P<0.02 by Mann–Whitney rank sum test). Error bar indicate s.e. (**f**) Representative morphology of mitochondria in shMock- and shMcu-expressing neurons. The enlarged right panels are straightened images from the dotted boxes. Scale bar, 5 μm. (**g**) Average mitochondrial length following treatment with shMock- (1,034 mitochondria) and shMcu-expressing neurons (1,130 mitochondria). (***P<0.01 by Mann–Whitney rank sum test).

Previous reports have shown that intra-mitochondrial Ca^2+^ entry is involved in mitochondrial division, although precise molecular mechanism was yet unclear^38^,^39^,^40^,^41^. Since CoMIC occurs before mitochondrial division and is mediated by intra-mitochondrial Ca^2+^, we tested the Ca^2+^-mediated functional link between CoMIC and mitochondrial division. First, we monitored the level of intra-mitochondrial Ca^2+^ during CoMIC and subsequent mitochondrial division in control neurons. Similarly to DN-Drp1-expressing neurons, control neuron also exhibited spontaneous, transient and dramatic increment of intra-mitochondrial Ca^2+^ just before and during CoMIC, and it returned to basal level during off-phase (Fig. 6a–c). However, there was no remarkable fluctuation of intra-mitochondrial Ca^2+^ during mitochondrial division. These data indicate that intra-mitochondrial Ca^2+^ is involved in induction of CoMIC but not Drp1-mediated mitochondrial division. Next, we examined the effect of intra-mitochondrial Ca^2+^ on mitochondrial length. Treatment with Ru360 significantly increased mitochondrial length and attenuated TG-induced mitochondrial fragmentation (Fig. 6d,e). Consistently, depletion of Mcu also significantly decreased mitochondrial length (Fig. 6f,g). These data indicate that intra-mitochondrial Ca^2+^ entry promotes mitochondrial division as well as CoMIC. Considering that mitochondrial division does not always accompany with fluctuation of intra-mitochondrial Ca^2+^, we suggest that intra-mitochondrial Ca^2+^ indirectly promotes mitochondrial division by promoting CoMIC.

### Mitochondrial bulging and depolarization is mediated by K^+^

**Figure 7:**
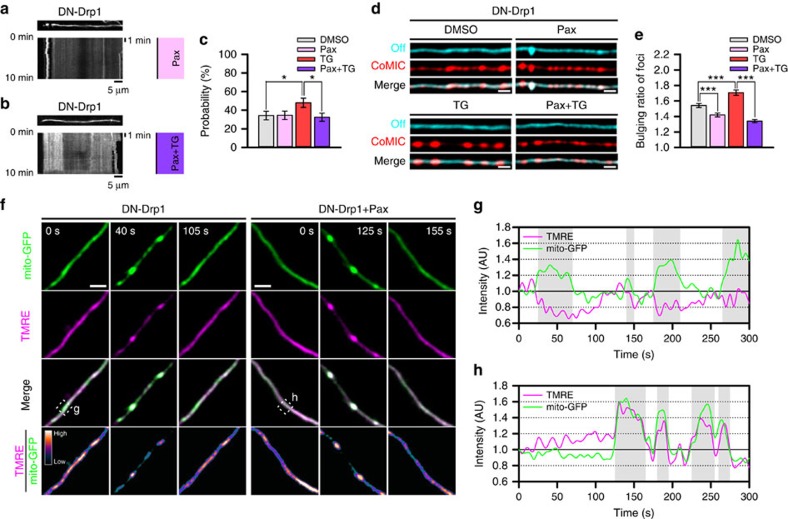
Intra-mitochondrial K^+^ influx is involved in mitochondrial bulging and depolarization during CoMIC. (**a**,**b**) Representative kymographies of DsRed-mito and patterns of CoMIC (right) in DN-Drp1-expressing neurons after treatment with paxilline (Pax, 10 μM) (**a**) and TG with 30 min pretreatment with Pax (Pax+TG) for 1 h (**b**). (**c**) Probability of CoMIC in elongated mitochondria treated with DMSO (103 mitochondria), Pax (110 mitochondria), TG (100 mitochondria) and Pax+TG (117 mitochondria) (*P<0.05 by Mann–Whitney rank sum test). Error bar indicate s.e. (**d**) Straightened images of DsRed-mito showing minimal (cyan signal) and maximal bulging (red signal). (**e**) Quantification of bulging ratio in foci stained with DsRed-mito (***P<0.01 by Mann–Whitney rank sum test). (**f**) Time-lapse images of mitochondria labelled by mito-GFP and TMRE in DN-Drp1-expressing neurons without or with Pax treatment. Scale bar, 5 μm, and TMRE/mito-GFP is pseudo-coloured. (**g**,**h**) Temporal fluctuations of TMRE and mito-GFP in dotted box of **f**. Gray boxes indicate CoMIC duration of mitochondria.

Mitochondrial bulging or swelling is induced by osmotic movement of water molecules into the mitochondrial matrix, and this process is mainly regulated by K^+^ influx controlled by the mitochondrial big-conductance Ca^2+^-dependent K^+^ channel (mitoBK_Ca_) and ATP-dependent K^+^ channel (mitoK_ATP_), which finally leads to an increase in osmotic pressure between the cytoplasm and the mitochondrial matrix^19^. Considering that intra-mitochondrial Ca^2+^ induces CoMIC accompanied by mitochondrial bulging, we investigated the possible involvement of mitoBK_Ca_ in CoMIC. Pre-treatment with paxilline (Pax), an inhibitor of mitoBK_Ca_, suppressed the probability of TG-induced, but not basal, CoMIC (Fig. 7a–c), but it did not affect the total duration, frequency or mean interval of CoMIC (Supplementary Fig. 12), implying partial involvement of induction/potentiation of CoMIC unlike Ca^2+^. Instead, Pax efficiently prevented basal and TG-induced mitochondrial bulging during CoMIC (Fig. 6d,e). Previous studies have demonstrated that intra-mitochondrial influx of K^+^ induces depolarization of mitochondria as well as bulging^42^,^43^. Recently, it has been also demonstrated that CoMIC accompanies with mitochondrial depolarization^23^,^24^. Consistently with these studies, we also found that mitochondria are transiently depolarized during CoMIC (Fig. 6f,g). Remarkably, Pax efficiently alleviated mitochondrial depolarization during CoMIC (Fig. 6f,h). On the other hand, prolonged treatment of NS1619, an opener of mitoBK_Ca_, induced robust CoMIC and eventual mitochondrial fragmentation in DN-Drp1-expressing neuron (Supplementary Fig. 13), although the effect of NS1619 may not be a result from mitoBK_Ca_ opening^44^. Collectively, these data indicate that mitoBK_Ca_ is involved in mitochondrial bulging and depolarization during CoMIC.

### Cleavage of Opa1 synergistically regulates CoMIC with Ca^2+^

**Figure 8:**
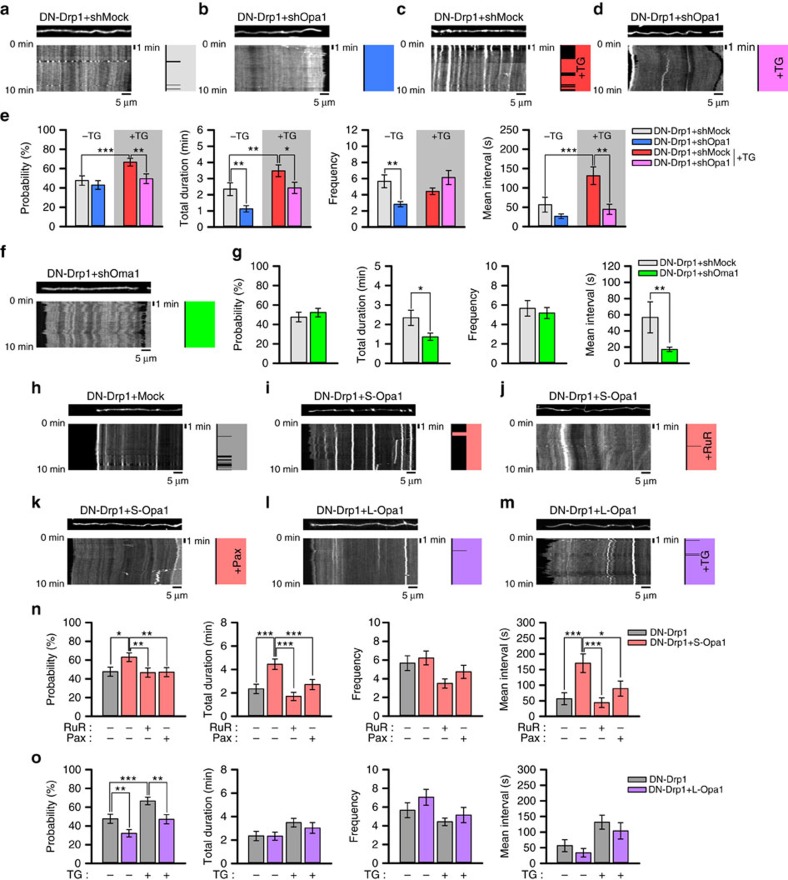
Oma1-mediated cleavage of Opa1 regulates CoMIC. (**a**–**d**) Representative kymographies of DsRed-mito and patterns of CoMIC (right diagram) in DN-Drp1+shMock- (**a**), DN-Drp1+shOpa1- (**b**), TG-treated DN-Drp1+shMock- (**c**) or TG-treated DN-Drp1+shOpa1-expressing neurons (**d**). (**e**) Probability of CoMIC in elongated mitochondria from DN-Drp1+shMock-, DN-Drp1+shOpa1- (123 mitochondria), TG-treated DN-Drp1+shMock- (138 mitochondria) and TG-treated DN-Drp1+shOpa1-expressing neurons (105 mitochondria), and quantification of the total duration, frequency and average single duration of CoMIC (***P<0.01, **P<0.02 and *P<0.05 by Mann–Whitney rank sum test). Error bar indicate s.e. (**f**) Representative kymography of DsRed-mito and patterns of CoMIC (right diagram) in DN-Drp1+shOma1-expressing neuron. (**g**) Probability of CoMIC in elongated mitochondria from DN-Drp1+shMock- and DN-Drp1+shOma1-expressing neurons (134 mitochondria), and quantification of the total duration, frequency and average single duration of CoMIC (**P<0.02 and *P<0.05 by Mann–Whitney rank sum test). (**h**–**m**) Representative kymographies of DsRed-mito and patterns of CoMIC (right diagram) in DN-Drp1- (**h**), DN-Drp1+S-Opa1- (**i**), ruthenium Red (RuR, 10 μM)-treated DN-Drp1+S-Opa1- (**j**), Pax-treated DN-Drp1+S-Opa1- (**k**), DN-Drp1+L-Opa1- (**l**) and TG-treated DN-Drp1+L-Opa1-expressing neurons (**m**). (**n**) Probability of CoMIC in elongated mitochondria from DN-Drp1- (105 mitochondria), DN-Drp1+S-Opa1- (111 mitochondria), RuR-treated DN-Drp1+S-Opa1- (105 mitochondria) and Pax-treated DN-Drp1+S-Opa1-expressing neurons (104 mitochondria), and quantification of the total duration, frequency and average single duration of CoMIC (***P<0.01, **P<0.02 and *P<0.05 by Mann–Whitney rank sum test). (**o**) Probability of CoMIC in elongated mitochondria from DN-Drp1-, TG-treated-DN-Drp1- (138 mitochondria), DN-Drp1+L-OPA1- (140 mitochondria) and TG-treated DN-Drp1+L-Opa1-expressing neurons (104 mitochondria), and quantification of the total duration, frequency and average single duration of CoMIC. (***P<0.01 and **P<0.02 by Mann–Whitney rank sum test).

Recently, Opa1 has been reported as a coupler between mitochondrial morphology and transient mitochondrial depolarization during CoMIC^24^. Because we found that mitochondrial Ca^2+^ rise is required, but not sufficient for CoMIC (Fig. 5a), we wondered a possible role of Opa1 in CoMIC. First, we knocked down the expression of Opa1 with shRNA (shOpa1), and found that shOpa1 efficiently inhibits CoMIC without affecting DN-Drp1-induced mitochondrial hyper-elongation (Fig. 8a,b and Supplementary Fig. 14). Although the probability of CoMIC was not affected, the total duration and frequency of CoMIC were significantly decreased by shOpa1 (Fig. 8e). In addition, shOpa1 significantly reduced the TG-increased probability of CoMIC as well as total duration and average single duration (Fig. 8c-e). These data indicate that Opa1 is required for induction and potentiation of CoMIC. We further examined whether other mitochondrial fusion machinery, Mfn1 and Mfn2, is also involved in CoMIC. Single depletion of Mfn1 or Mfn2 by shRNA affected neither DN-Drp1-potentiated CoMIC nor mitochondrial morphology (Supplementary Fig. 6). Notably, double depletion of Mfn1 and Mfn2 strongly inhibited DN-Drp1-induced mitochondrial hyper-elongation, thereby reducing CoMIC, which occurs in only elongated mitochondria. However, elongated mitochondria also exhibited DN-Drp1-potentiated CoMIC, and it appears that mitochondrial fusion indirectly contributes to potentiation of CoMIC by elongating mitochondria.

It has been known that IMM bound long Opa1 (L-Opa1) is cleaved by Oma1, which senses mitochondrial depolarization, resulting in the accumulation of soluble short Opa1 (S-Opa1) in the intermembrane space^45^. Thus, we postulated that Oma1 is upstream regulator of Opa1 in CoMIC. Very similarly to shOpa1, depletion of Oma1 by shRNA significantly reduced, but not probability, the total and average single duration of CoMIC without modifying DN-Drp1-induced mitochondrial hyper-elongation (Fig. 8f,g and Supplementary Fig. 15). It indicates that Oma1-mediated cleavage of Opa1 may play a role in the regulation of CoMIC. Recently, it has been demonstrated that S-Opa1 can induce mitochondrial fragmentation and its GTPase-defective mutant localizes to the foci of constriction and division with Drp1 at the ER–mitochondria contact site^46^. Thus, we further examined the differential involvement of the S-Opa1 and L-Opa1 in CoMIC. We co-transfected neurons with S-Opa1 fusing intermembrane space-localizing signal of apoptosis-inducing factor or L-Opa1 lacking cleavage sites with DN-Drp1 (Supplementary Fig. 16a). While S-Opa1 induced excessive mitochondrial fragmentation in neurons and 293T cells as shown in previous report^46^, its co-expression with DN-Drp1 did not affect mitochondrial hyper-elongation by DN-Drp1 (Supplementary Fig. 16b,c), indicating that S-Opa1-induced mitochondrial fragmentation requires Drp1. Interestingly, in neurons co-expressing S-Opa1 with DN-Drp1, a greater proportion of mitochondria exhibited CoMIC-like structures (Fig. 8i). Quantitative analyses showed that the probability, total duration and average single duration of CoMIC were dramatically increased by S-Opa1 (Fig. 8h,i,n), indicating that the S-Opa1 sufficiently promotes induction/potentiation of CoMIC. In contrast, L-Opa1 induced mitochondrial elongation in control neurons, but it did not affect in DN-Drp1-expressing neuron (Supplementary Fig. 16b,c). Notably, L-Opa1 exhibited inhibitory effects on the induction of basal CoMIC and TG-induced CoMIC (Fig. 8l,m,o), indicating inhibitory role of L-Opa1 in CoMIC.

Interestingly, the effect of S-Opa1 was efficiently blocked by ruthenium Red (RuR), an inhibitor of mitochondrial calcium uptake, or Pax (Fig. 8j,k,n). These data suggest that S-Opa1-induced/potentiated CoMIC requires intra-mitochondrial influx of Ca^2+^/K^+^. Taken together, these data suggest that intra-mitochondrial influx of Ca^2+^/K^+^ and cleavage of Opa1 are two interdependent pathways for the regulation of CoMIC.

### Opa1 regulates OMM-IBM tethering during CoMIC

**Figure 9:**
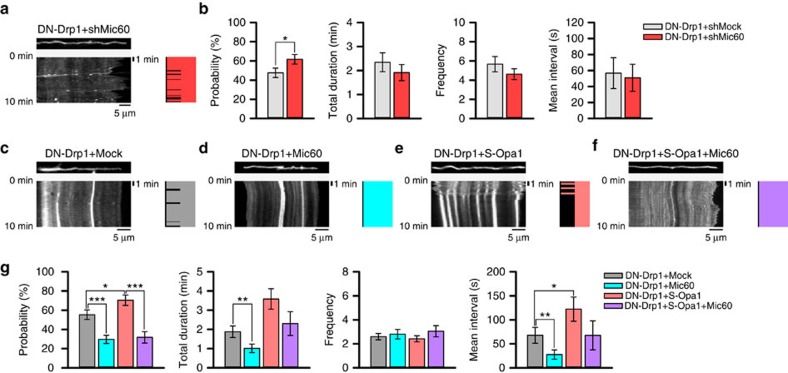
Opa1 neutralizes Mic60-mediated tethering OMM and IMM so regulating CoMIC. (**a**) Representative kymography of DsRed-mito and patterns of CoMIC (right diagram) in DN-Drp1+shMic60-expressing neurons. (**b**) Probability of CoMIC in elongated mitochondria from DN-Drp1+shMock- and DN-Drp1+shMic60-expressing neurons (94 mitochondria), and quantification of the total duration, frequency and average single duration of CoMIC (*P<0.05 by Mann–Whitney rank sum test). Error bar indicate s.e. (**c**–**f**) Representative kymographies of mito-GFP and patterns of CoMIC (right diagram) in DN-Drp1+Mock (**c**), DN-Drp1+Mic60- (**d**), DN-Drp1+S-Opa1- (**e**) and DN-Drp1+S-Opa1+Mic60-expressing neurons (**f**). (**g**) Probability of CoMIC in elongated mitochondria from DN-Drp1-, DN-Drp1+Mic60- (118 mitochondria), DN-Drp1+S-Opa1- (71 mitochondria) and DN-Drp1+S-Opa1+Mic60-expressing neurons (63 mitochondria), and quantification of the total duration, frequency and average single duration of CoMIC (***P<0.01, **P<0.02 and *P<0.05 by Mann–Whitney rank sum test).

**Figure 10:**
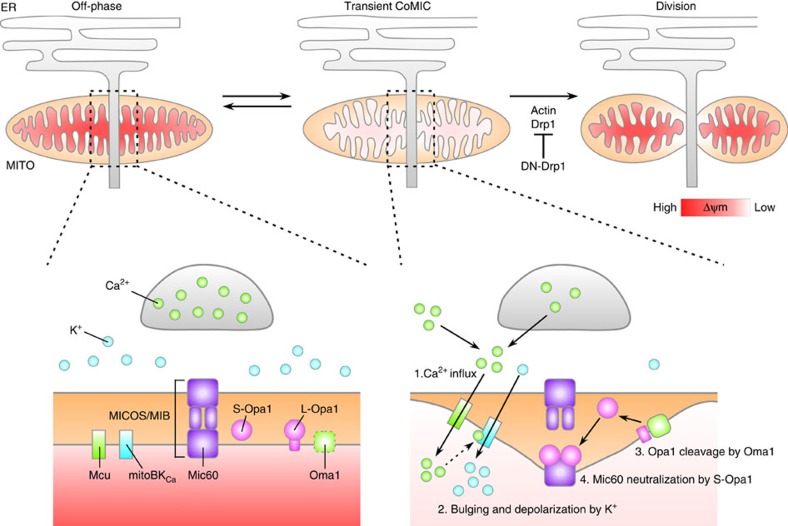
Schematic model of CoMIC for mitochondrial division. Intra-mitochondrial Ca^2+^ flux triggers mitoBK_Ca_-mediated mitochondrial bulging and depolarization. Synergistically, stabilized Oma1 cleaves L-Opa1 resulting accumulation of S-Opa1, which neutralizes Mic60-mediated OMM–IBM tethering. This CoMIC contributes to Drp1-mediated mitochondrial division.

Finally, we explored how S-Opa1 regulates CoMIC. S-Opa1 is a component of mitochondrial contact site and intermembrane space bridging (MIB) complex, which play a role in cristae morphogenesis and mitochondrial inner boundary membrane (IBM)–OMM tethering, together with Mic60 (Mitofilin/Immt), Mic19 (ChchD3), Mic25 (ChchD6) and Sam50 (refs 47, 48). Based on the rearrangement of OMM–IBM during CoMIC (Fig. 4), we postulated the involvement of Mic60 in the regulation of CoMIC by S-Opa1. Remarkably, Mic60 depletion by shRNA significantly increased probability of CoMIC in DN-Drp1-expressing neurons (Fig. 9a,b and Supplementary Fig. 17a), although it did not affect DN-Drp1-induced mitochondrial hyper-elongation (Supplementary Fig. 17b,c). Conversely, Mic60 overexpression strongly blocked the induction and potentiation of CoMIC in DN-Drp1-expressing neurons (Fig. 9c,d and Supplementary Fig. 17a,d,e). Quantitative analyses showed that the probability, total duration and mean interval of CoMIC are markedly decreased by Mic60 overexpression (Fig. 9g). Co-expression of Mic60 with S-Opa1 in DN-Drp1-expressing neuron remarkably inhibited S-Opa1-induced/potentiated CoMIC (Fig. 9e–g), suggesting that Mic60 is inactivated by S-Opa1 and the S-Opa1-induced CoMIC can be reversed by overexpression of Mic60 (Fig. 10).

## Discussion

In this study, we found that a subset of elongated mitochondria underwent transient and repetitive CoMIC, which was closely associated with subsequent mitochondrial division. So far, CoMIC-like events have been proposed to be associated with mitochondrial physiologies, including mitochondrial uncoupling, ROS production and oxidative phosphorylation^22^,^23^,^24^. In this study, we suggest that CoMIC is strongly associated with mitochondrial division, based on that (1) mitochondria undergoing CoMIC have higher probability for subsequent mitochondrial division; (2) mitochondrial division occurs at the CoMIC foci; (3) suppression of Drp1 strongly influences the extent of CoMIC; and (4) conditions blocking CoMIC suppressed mitochondrial division. Furthermore, by inhibiting mitochondrial division, we dissected intra-mitochondrial events mediating the CoMIC; (1) intra-mitochondrial Ca^2+^ flux triggering CoMIC, (2) mitoK_Ca_-mediated mitochondrial bulging and depolarization and (3) Oma1-mediated accumulation of S-Opa1, which neutralizes Mic60-mediated OMM–IBM tethering (Fig. 10). Therefore, we propose that CoMIC is an intra-mitochondrial priming event for mitochondrial division.

Although we primarily focused on the mitochondria in neuronal processes, CoMIC was also found in multiple cell types including fibroblasts (mesoderm-derived cells) and HepG2 cells (endoderm-derived cells) as well as neurons (ectoderm-derived cells). Previous reports have also showed CoMIC-like mitochondrial constriction in other many mammalian cells under normal and pathologic conditions^22^,^23^,^24^,^25^,^49^,^50^,^51^,^52^. Furthermore, this event has also been observed in yeast^13^, *C. elegans*^14^ and even plants^53^. Therefore, we suggest that the fundamental machinery triggering CoMIC may be conserved and operating in most, if not all, eukaryotic cells. Mitochondrial influx of Ca^2+^ plays an essential role in the induction of CoMIC. Interestingly, Ca^2+^ also promotes cellular constriction in prokaryotic cells by enhancing FtsZ polymerization and further bundling of FtsZ polymers^54^. Although mammalian FtsZ ring does not exist, this implies that Ca^2+^-mediated CoMIC seems to be a fundamental and highly conserved machinery from prokaryotic to eukaryotic cells.

Controversial data reported that there was no detectable change in intra-mitochondrial Ca^2+^ concentration during a CoMIC-like event^23^. However, we used more sensitive genetic dye CEPIA3*mt* (ref. 37), and could detect rise on intra-mitochondrial Ca^2+^ during CoMIC. In addition, Ru360 and depletion of Mcu inhibited DN-Drp1-potentiated CoMIC. Considering that elongated mitochondria can more efficiently absorb Ca^2+^ than short mitochondria in neuronal cells^55^, mitochondrial hyper-elongation by DN-Drp1 may amplify intra-mitochondrial Ca^2+^ signals. When Drp1 is expressed normally and mitochondria is endogenously elongated, CoMIC is very transient and the effect of intra-mitochondrial Ca^2+^ influx on CoMIC may be small enough to be virtually undetectable. Furthermore, we showed that blockade of intra-mitochondrial Ca^2+^ entry inhibits efficient mitochondrial division. Consistently with our data, previous pharmacological studies have shown that Ru360 inhibits mitochondrial fragmentation by Ca^2+^ released from the ER by p20, which is a cleaved form of BAP31 by caspase 8 during Fas-mediated cell death^39^. Furthermore, inhibition of mitochondrial Ca^2+^ uptake by NIM811 and carbonyl cyanide-p-trifluoromethoxyphenyl-hydrazone, which are a mitochondrial permeability transition inhibitor and a mitochondrial uncoupler, also blocks mitochondrial fragmentation by NMDA-mediated entry of extracellular Ca^2+^ in neurons^39^,^41^,^52^,^56^. Conversely, cells with mutations in the *Micu1* gene, which regulates Mcu activity, exhibit excessively fragmented mitochondria with increased intra-mitochondrial Ca^2+^ (ref. 57). These reports indicate that intra-mitochondrial Ca^2+^ is critical for efficient mitochondrial division. However, considering that mitochondrial division did not directly accompany intra-mitochondrial Ca^2+^ rise, Ca^2+^-mediated CoMIC is a prior event before mitochondrial division. In fact, intracellular Ca^2+^ activates cytosolic vesicle-forming machinery for mitochondrial division. Drp1 is upregulated by the Ca^2+^-dependent kinase CaMKIα following neuronal depolarization^58^ or the Ca^2+^-dependent phosphatase calcineurin following mitochondrial damage and neuronal outgrowth^59^,^60^. In addition, Ca^2+^ can activate inverted formin-2, thereby promoting actin polymerization^61^. Therefore, Ca^2+^ mediates and coordinates both of cytosolic and intra-mitochondrial events for mitochondrial division. According to this model, a substantial increase in cytosolic Ca^2+^ can recruit Drp1 to the mitochondria to complete mitochondrial division, whereas CoMIC occurs when a lower level of Ca^2+^ is released from the ER and taken up into mitochondria.

Our data revealed that mitochondrial bulging during CoMIC is mediated by K^+^ via mitoBK_Ca_. Because the concentration of K^+^ in the cytosol is higher than that in mitochondria, as the IMM is impermeable to potassium ions, mitochondrial influx of K^+^ across the IMM requires specific channels, mitoBK_Ca_ and mitoK_ATP_^19^. Opening of these channels induces mitochondrial influx of K^+^, resulting in osmotic movement of water molecules into the mitochondrial matrix, thereby causing mitochondrial bulging or swelling. Potential role of intra-mitochondrial K^+^ in mitochondrial volume and morphology has been elucidated. Intra-mitochondrial influx of K^+^ promotes the formation of doughnut-like mitochondria during mitochondrial adaptation to conditions of hypoxia-reoxygenation^62^, and is also involved in formation of CoMIC-like structures by depletion of Letm1, which regulates homoeostasis of intra-mitochondrial Ca^2+^ and K^+^ (ref. 63). Furthermore, inhibition of aquaporin by HgCl_2_ suppresses tunicamycin-induced CoMIC and further mitochondrial fragmentation^64^, indicating the involvement of osmosis in mitochondrial division. Notably, the Ca^2+^-responsive regulatory domain of mitoBK_Ca_ is likely to face the mitochondrial matrix rather than the cytosol^65^, supporting the notion that intra-mitochondrial Ca^2+^, but not cytosolic Ca^2+^, mediates mitochondrial bulging. In addition, we found that DN-Drp1 enhances mitochondrial bulging during CoMIC, and it appears to result from increased Ca^2+^ uptake by mitochondrial hyper-elongation^55^. Although previous report demonstrated that H^+^ leak contributes to mitochondrial depolarization during CoMIC^30^,^31^, we found that inhibition of mitoBK_Ca_ efficiently suppresses mitochondrial depolarization during CoMIC. Recent study demonstrated the transient mitochondrial pH flash by bulky H^+^ leakage, which could be induced by intra-mitochondrial Ca^2+^/K^+^ influx^66^. Alternatively, the H^+^ leak may contribute to recovery from CoMIC to off-phase, because efflux of Ca^2+^ and K^+^ from the mitochondrial matrix are mainly mediated by a mitochondrial Na^+^/Ca^2+^ exchanger and K^+^/H^+^ exchanger driven by H^+^ leak^19^. However, we also found that long exposure to carbonyl cyanide *m*-chlorophenyl hydrazone (CCCP), which disrupts the proton gradient and induces bulky H^+^ leak, induces irreversible CoMIC with mitochondrial depolarization (Supplementary Fig. 18). Therefore, although we could not completely exclude that H^+^ leak may mediate CoMIC, it appears that intra-mitochondrial Ca^2+^/K^+^ influx mainly mediates morphological and physiological change during CoMIC.

Our data also suggested that Oma1-induced S-Opa1 induces and potentiates CoMIC. Recent studies have highlighted the role of S-Opa1 on mitochondrial division. Unlike L-Opa1, S-Opa1 sufficiently induced Drp1-dependent mitochondrial fragmentation in neurons, in agreement with a previous report^46^. Considering that potentiation of CoMIC in DN-Drp1-expressing neurons was further strengthened by overexpression of S-Opa1, it is likely that S-Opa1 promotes CoMIC. However, shOpa1 did not influence the basal probability of CoMIC, although it efficiently suppressed the frequency and duration of CoMIC. Therefore, it is likely that S-Opa1 does not directly constrict the IMM, but is involved in the regulation of CoMIC. Based on our data with previous studies, S-Opa1 may have pro-fission activity. However, S-Opa1 is required for proper mitochondrial fusion^67^, and *in vitro* IMM fusion can be promoted by S-Opa1 produced by i-AAA protease Yme1l under active oxidative phosphorylation and by Oma1 under mitochondrial depolarization^46^,^68^. This opposite effect of S-Opa1 could be mediated by disorganization of OMM–IBM contact via interplay between S-Opa1 and Mic60. The effect of S-Opa1 on the CoMIC was strongly modified by alterations of Mic60, which plays a role in tethering of OMM–IBM as core component of mitochondrial contact site and MIB complex^47^,^48^. Mic60 is evenly localized in IMM^69^, but S-Opa1 is preferentially localized in MIB complex and interacts with Mic60 (refs 47, 48). Considering that S-Opa1 and L-Opa1 form oligomer^70^, this model may explain how L-Opa1 inhibits CoMIC: L-Opa1 may inhibit the S-Opa1- and Mic60-mediated activation of CoMIC via neutralization of S-Opa1 as a dominant-negative molecule. Therefore, we propose that S-Opa1 is involved in the disorganization of OMM–IBM, whereas L-Opa1 antagonizes this process, thereby contributing to the CoMIC and IMM reorganization.

It is likely that intra-mitochondrial Ca^2+^–K^+^ and Oma1–Opa1–Mic60 are two different axes for the control of CoMIC, and both are required for the efficient CoMIC. Therefore, inhibition of either cascades appear to inhibit CoMIC, while we do not entirely rule out the possibility that two cascades have significant interactions. For example, intra-mitochondrial influx of Ca^2+^ induces mild depolarization of mitochondria, leading to Oma1-dependent cleavage of Opa1 (refs 40, 46). On the other hand, Opa1 can also regulate intra-mitochondrial influx of Ca^2+^. In digitonin-permeabilized cells, depletion of Opa1 increases intra-mitochondrial influx of Ca^2+^ in spite of a dissipation of the mitochondrial membrane potential^71^, indicating that Opa1 plays a role in intra-mitochondrial structure for proper calcium uptake. Therefore, mutually dependent interaction between Ca^2+^ and Opa1 could be an important aspect explaining the cyclic nature of CoMIC, and the blockade of any part of this cascade can disrupt the CoMIC cycle.

## Methods

### Cell culture and gene transfection

Cortices were carefully dissected from Sprague–Dawley rats (Orient Bio and Koatech, Korea) on embryonic day 17 in pre-chilled Hank's buffered salt solution (Gibco) supplemented with 6% glucose (Sigma) and 50 unit per ml penicillin–streptomycin (PS) (Gibco). Then, the samples were trypsinized and physically dissociated into single neurons. The dissociated neurons (10^5^ cells per cm^2^) were plated on glass coverslips, plates and dishes coated with poly-D-lysine (sigma), and incubated in neurobasal media (Gibco) containing 2% B27 supplement (Gibco), 0.5 mM L-glutamine (Gibco), 25 μM L-glutamate (Sigma) and 50 unit per ml penicillin/streptomycin (Gibco). The neurons were incubated in 5% CO_2_ at 37 °C. This experiment was carried out in strict accordance with the recommendations in the Guide for the Care and Use of Laboratory Animals of the Korea University Institutional Animal Care and Use Committee. The protocol was approved by the Committee on the Ethics of Animal Experiments of the Korea University (Permit Number: KUIACUC20110304-2). After 2 days *in vitro* (DIV2), we exchanged the medium for media containing no L-glutamate and maintained the cultured neurons in this medium. We transfected plasmids into cultured neurons on DIV2 or DIV8 using the Calphos mammalian transfection kit (Clontech) according to the manufacturer's protocol. After 24–48 h (DIV3–4 or DIV9–10), experiments were performed. Thapsigargin, Ru360 and latrunculin B were purchased from Calbiochem. Paxilline and CCCP were purchased from Abcam. Two-APB, ruthenium red and mito-TEMPO were purchased from Sigma. Drp1-knockout mouse embryonic fibroblasts were obtained from Dr Hiromi Sesaki^17^. 293T (ATCC CRL-3216), HeLa (ATCC CRM-CCL-2) and HepG2 (ATCC HB-8065) cells were incubated with Dulbecco's modified eagle medium (Gibco) supplemented with 10% foetal bovine serum (Gibco) and 50 unit per ml penicillin/streptomycin in 5% CO_2_ at 37 °C, and gene transfection was performed using Lipofectamine 2000 (Invitrogen, USA) and the Calphos mammalian transfection kit according to the manufacturer's protocols. Experiments were performed 24 h after transfection.

### DNA constructs

DsRed-mito and mito-GFP, which label the mitochondrial matrix, were purchased from Clontech. The PCR product of DN-Drp1 was obtained from pcDNA-Drp1 (K38A)^28^ and inserted using the XhoI (5′) and EcoRI (3′) sites of pIRES2-GFP (Clontech) and pIRES-DsRed-mito, which was modified from pIRES2-GFP by exchanging GFP with DsRed-mito. Tom20-GFP was generated by insertion of a human *Tom20* PCR fragment into the XhoI (5′) and SalI (3′) sites of pEGFP-N1 (Clontech). GFP-Sec61b was generated by insertion of human *Sec61b* PCR fragment into the XhoI (5′) and EcoRI (3′) sites of pEYFP-C1 (Clontech). Via serial PCR, a flag-tagged S-Opa1 fragment was obtained by fusing the N-terminal signal peptide of apoptosis-inducing factor (amino acids 1–95) and the C-terminal of the human *Opa1* isoform 1 cDNA (amino acids 202–969). A flag-tagged L-Opa1 lacking proteolytic cleavage sites (amino acids 191–201) also was constructed by serial PCR. These DNA fragments were inserted into the BamHI (5′) and XhoI (3′) sites of pcDNA3.0 (Invitrogen, USA). In addition, myc-tagged *Mic60* and *Oma1* cDNAs were obtained by RT–PCR from RNA of rat cultured neuron, and were inserted into the BamHI (5′) and XhoI (3′) sites of pcDNA3.0. GFP-Mff (Addgene plasmid # 49153) and mito-BFP (Addgene plasmid # 49151) were gifts from Gia Voeltz. CEPIA3*mt* (Addgene plasmid # 58219) and Twinkle–GFP also were gifts from Masamitsu Iino and Johannes N. Spelbrink, respectively.

The targeted sequences for shRNA were 5′-GAA GAG TGT AAC TGA TTC A-3′ for rat *Drp1*, 5′-GCC AGA GAC AGA CAA TAC T-3′ for rat *Mcu*, 5′-GAT TGT GCC TGA CTT TAT A-3′ for rat Opa1, 5′-CGA AAC CAG ATG AAC CTT T-3′ for rat *Mfn1*, 5′-TGA GGA TGT TTG AGT TTC A-3′ for rat *Mfn2*, 5′-GCC ATA AGA GAG GTC CGG A-3′ for rat *Oma1* and 5′-CTG AGA TTG CAG GTG AGA A-3′ for rat *Mic60*. The shRNA constructs were generated using the pSuper.neo+GFP vector (OligoEngine) according to the manufacturer's protocol.

### Immunoblotting

Rat cortical neuron and 293T cell were collected in lysis buffer (125 mM Tris-Cl, 4% SDS, pH 6.8). After SDS–PAGE and transfer into membrane, immunoblotting was performed using the following antibodies: anti-Opa1 (BD Transduction Laboratories, #612606, 1:500), anti-Mcu (abcam, #ab121499, 1:1,000), anti-myc (Santa Cruz Biotechnology, #sc-40, 1:1,000) and anti-ß-Actin (Santa Cruz Biotechnology, #sc-47778, 1:2,000).

### Live cell imaging and microscope image acquisition

Live cell imaging was mainly performed using an inverted fluorescence microscope (Carl Zeiss, Observer Z1) equipped with CoolLED (pE-2) as the light source, a definite focus module, a CCD camera (Photometrics, CoolSNAP fx), and a humidified chamber that maintained cells at 5% CO_2_ and 37 °C. Primary neurons and other cell lines were incubated in the appropriate culture media during live cell imaging. A × 63 oil-immersion objective lens (Carl Zeiss, EC Plan-Neofluar, 1.25 NA) was used for image acquisition, and time-lapse images were obtained every 5 s for 10 min using the Metamorph (version 7.7.5) imaging program (Molecular Devices). In addition, we used a confocal inverted laser-scanning microscope (Carl Zeiss, LSM7 LIVE) equipped with a humidified incubator that maintained cells in 5% CO_2_ at 37 °C, and images were obtained with ZEN software (Carl Zeiss) with a × 63 oil-immersion objective lens (Carl Zeiss, Plan-Apochromat, 1.4 NA). High-resolution live cell confocal images were obtained using an inverted laser-scanning microscope (Carl Zeiss, DE/LSM510 NLO) equipped with a humidified incubator that maintained cells in 5% CO_2_ at 37 °C. A × 100 oil-immersion objective lens (Carl Zeiss, EC Plan-Neofluar, 1.3 NA) was mainly used with × 3 digital zoom for image acquisition. Confocal serial sectional images were obtained every 10 s for 10 min using ZEN software (Carl Zeiss). Each time-lapse image consists of four or five sections obtained with a centred Z-section mode. For synchronized imaging of dual channels, we used two mixed lasers, 488 and 536 nm, for emission, and detected excitation using a splitter filter. To avoid excessive photo-damage caused by the LED or laser, we took time-lapse images from one slide within 1–1.5 h after drug treatment. The images were analyzed using the ImageJ program (USA National Institutes of Health). We linearly adjusted the intensity of the images, smoothed the images with ‘Gaussian Blur' filtering and reconstructed three-dimensional images using the ‘3D viewer' module. The reconstructed images were recorded in 360° rotation. For measurement of mitochondrial length, we first fixed primary neurons expressing DsRed-mito with 4% paraformaldehyde in PBS, then stained their nuclei with 1 μg ml^−l^ Hoechst 33342. Then, we captured images using an upright fluorescent microscope (Carl Zeiss Axioskop2 Plus) with a CCD camera (Photometrics, CoolSNAP cf) using the Metamorph imaging programme. A × 40 objective lens (Carl Zeiss, EC Plan-Neofluar, 0.75 NA) was used for image acquisition. In addition, we used a confocal inverted laser-scanning microscope (Carl Zeiss, LSM700). A × 40 water-immersion objective lens (Carl Zeiss, LD C-Apochromat, 1.1 NA) was mainly used with × 3 digital zoom for image acquisition. FRET images were acquired by confocal microscopy (Carl Zeiss, LSM700) using microscope filter combination for BFP and FRET: 405 nm excitation, 0–415 nm and 415–735 nm emission filters, respectively. Images were analysed using ImageJ software.

### Kymographic and quantitative analyses of CoMIC

All images were modified with the ImageJ programme. For kymographic analysis, we modified time-lapse images of mitochondria in curved neuronal processes to straight images using the ‘straighten' function. Then, the images were resliced to *y* axis and Z-projected with the ‘reslice' and ‘Z-project' modules. We manually generated constriction spikes (right diagram of kymography). Each incidence of CoMIC was labelled as one spike, and the duration of the CoMIC was expressed by the thickness of the spike. We performed quantitative analyses, including the total duration, frequency and mean interval, of CoMIC from all mitochondria longer than 5 μm in neuronal processes ∼200 μm from the soma. To measure the bulging ratio of foci, we selected two mitochondrial images of off-phase and maximum bulging from more than 10 CoMIC-containing mitochondria, and measured the ratio of the diameter of each foci in the off-phase and bulging site.

### Measurement of mitochondrial length

Mitochondrial images captured from all neuronal processes (200 μm from the soma) were converted to binary images using the ‘threshold' module in ImageJ. Then, the binary images were converted to images 1 pixel wide by the ‘skeletonize' module, and mitochondrial length was assessed by the ‘analyse particles' module.

### Electron microscopy

The Drp1 knockdown viral constructs were transfected into 293gpg packaging cells using PEI (Polyscience, #23966). After transfection, media were changed to the neurobasal media containing 2% B27 supplement, 0.5 mM L-glutamine and 50 unit per ml penicillin/streptomycin. The supernatants containing viral particles were collected at 2–4 days after transfection, and infected primary cortical neurons. Drp1 knockdown cortical neurons were washed with 0.1 M phosphate-buffered saline (pH 7.4, PBS) and fixed with 10% paraformaldehyde, 2.5% glutaraldehyde in PBS at 4 °C for overnight. After fixation, cells were washed with PBS and post fixed with 1% osmium tetroxide for 90 min. The fixed cells were dehydrated through ascending series of ethanol, and then embedded in Epon mixture. The embedding blocks were semi-thin sectioned (200 nm) using a Reichert-Jung Ultracut E ultramicrotome (Leica Microsystems), stained with Toluidine blue and trimmed for further observation. The trimmed sections were collected on 200-mesh cooper grid and stained with uranyl acetate and lead citrate. Observation of mitochondrial structure was performed using an H-7500 electron microscope (Hitachi) with 80 kV acceleration voltage.

### Statistical analysis

Statistical analyses and graph plotting were conducted using the Sigmaplot 12.5 software. Centre values and error bars of all graph indicate mean value and s.e., respectively. To measure the probability of CoMIC, we separated mitochondria into those that showed CoMIC and those that did not, and assigned them values of 100% or 0%, respectively. Then, we averaged them and tested statistical significance with the Mann–Whitney rank sum test. To examine the statistical significance of the proportion of mitochondria undergoing subsequent division to that of mitochondria showing no or prior CoMIC, we generated contingency tables according to the presence of CoMIC and division. The contingency tables were tested with *χ*^2^-tests with Yates's correction for continuity. Similarly, contingency tables for mitochondria showing prior CoMIC among non-dividing or dividing mitochondria were also tested with a *χ*^2^-test with Yates's correction for continuity. For statistical analysis of total CoMIC duration, frequency and mean interval and bulging ratio of the foci containing CoMIC, the Mann–Whitney rank sum test was used.

### Data availability

The authors declare that all data supporting the findings of this study are available within the article and its Supplementary Information Files or from the corresponding author on reasonable request.

## Additional information

**How to cite this article:** Cho, B. *et al*. Constriction of the mitochondrial inner compartment is a priming event for mitochondrial division. *Nat. Commun.* **8,** 15754 doi: 10.1038/ncomms15754 (2017).

**Publisher's note:** Springer Nature remains neutral with regard to jurisdictional claims in published maps and institutional affiliations.

## Supplementary Material

Supplementary Information Supplementary Figures.

Supplementary Movie 1 A mitochondrion showing CoMIC and subsequent division. Mitochondria was monitored in cultured cortical neuron transfected with DsRed-mito (DIV10) by using an inverted fluorescence microscope (Germany, Carl Zeiss Observer Z1) equipped with CoolLED (pE-2) as the light source at 5% CO_2_ and 37°C. Images were taken per 5 seconds for 10 minutes, and below graph represents dynamic intensity of DsRed-mito signal.

Supplementary Movie 2 Enhanced CoMIC in DN-Drp1-expressing neuron. Cultured cortical neuron (DIV4) was transfected with DsRed-mito and DN-Drp1. By using an inverted fluorescence microscope (Germany, Carl Zeiss Observer Z1) equipped with CoolLED (pE-2) as the light source at 5% CO_2_ and 37°C, images of DsRed-mito were taken per 5 seconds for 10 minutes.

Supplementary Movie 3 Differential changes on mitochondrial matrix and OMM during CoMIC in DN-Drp1-expressing neuron. Cultured cortical neuron was transfected with DsRed-mito, hTom20-GFP and DN-Drp1 (DIV4). By using an inverted laser-scanning microscope (DE/LSM510 NLO, Carl Zeiss, Germany) at 5% CO_2_ and 37°C, images of DsRed-mito (red) and hTOM20-GFP (green) were taken at same time per 10 seconds for 10 minutes. Each image was Z-projected from four Z-sections. Below graphs represents dynamic intensities of DsRed-mito and hTom20-GFP.

Supplementary Movie 4 Spatial intersection between mitochondria and ER during CoMIC in DN-Drp1-expressing neuron. Cultured cortical neuron was transfected with DsRed-mito, YFP-hSec61b and DN-Drp1 (DIV4). By using an inverted laser-scanning microscope (DE/LSM510 NLO, Carl Zeiss, Germany) at 5% CO_2_ and 37°C, images of DsRed-mito (red) and YFP-hSec61b (green) were taken at same time per 10 seconds for 10 minutes. Each image was Z-projected from four Z-sections. Below graphs represents dynamic intensities of DsRed-mito and hSec61b-GFP.
