# Supplementary material for: Constriction of the mitochondrial inner compartment is a priming event for mitochondrial division
Source: Nat Commun. 2017 Jun 9;8:15754. doi: 10.1038/ncomms15754 (PMC5472732; doi:10.1038/ncomms15754)
Supplement: Supplementary Information — Supplementary Figures. [file ncomms15754-s1.pdf]

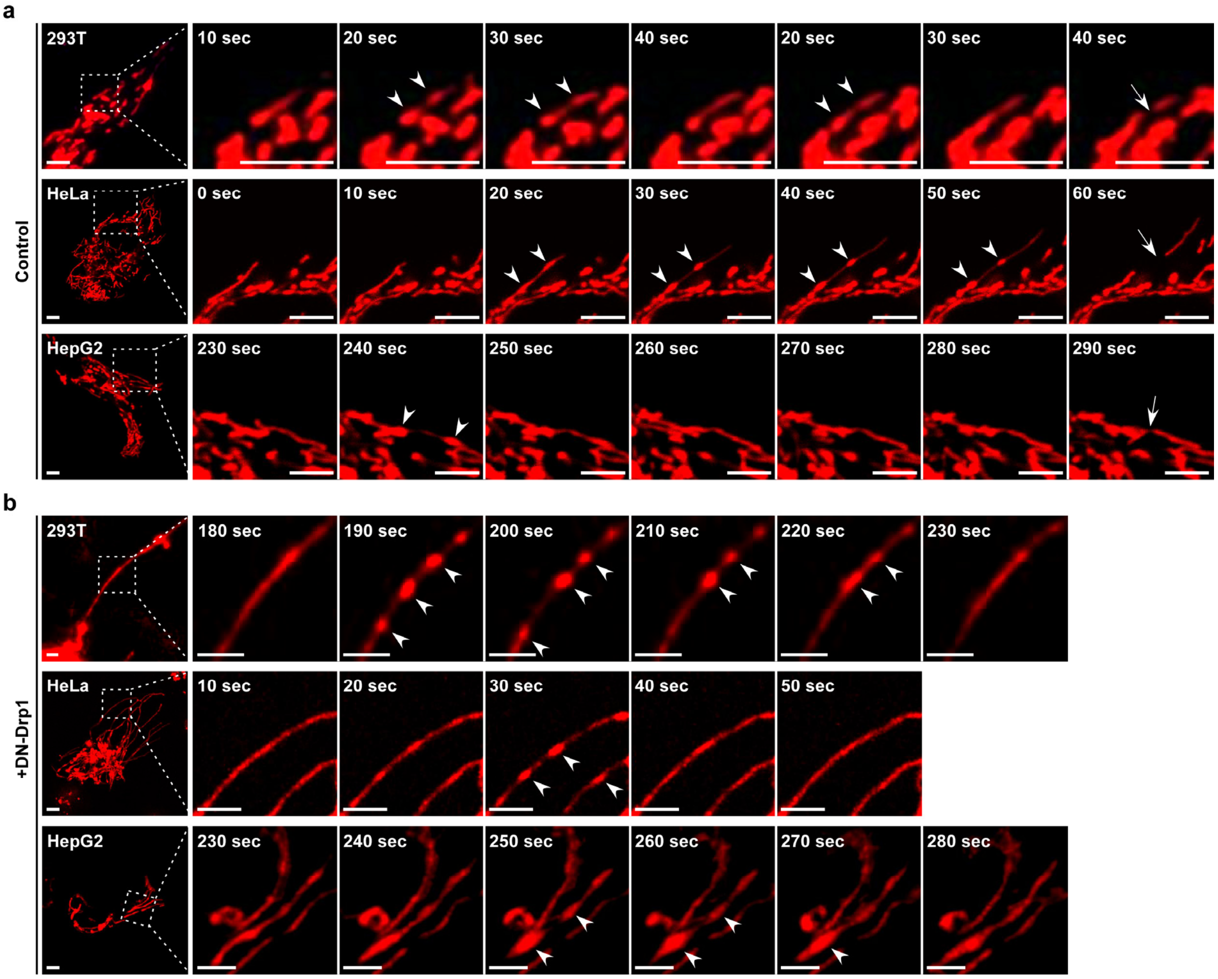

Supplementary Figure 1. CoMIC in 293T, HeLa, and HepG2 cells.

(**a**) Mitochondrial morphology in 293T, HeLa and HepG2 cells. Cells were transfected with DsRed-mito. Right panels are time-course enlarged images in insets. Arrow heads and arrows indicate mitochondrial bulging and division, respectively. (**b**) Mitochondrial morphology and time-course enlarged images in 293T, HeLa and HepG2 cells expressing DN-Drp1. All scale bars represent 5 µm.

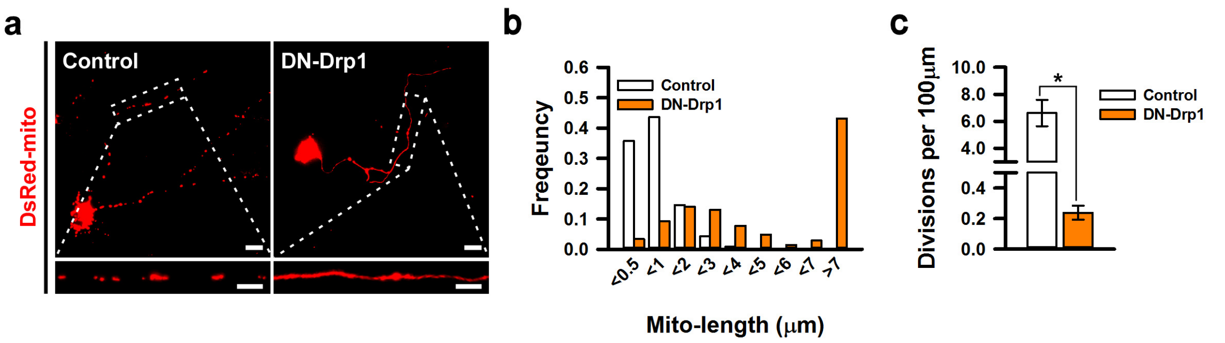

Supplementary Figure 2. Over-expression of DN-Drp1 induces hyper-elongation of mitochondria.
(**a**) Morphologies of mitochondria in control (left) or DN-Drp1-expressing neurons (right). Scale bars represent 10 μm. (**b**) Frequency-fractionation analysis of mitochondrial length. (Control, 601 mitochondria; DN-Drp1, 206 mitochondria) (**c**) Number of divisions per 100 μm of mitochondria during 10 min. (Control, 70 mitochondria; DN-Drp1, 180). (*p<0.05, by the Mann-Whitney rank sum test). Error bar indicates standard error.

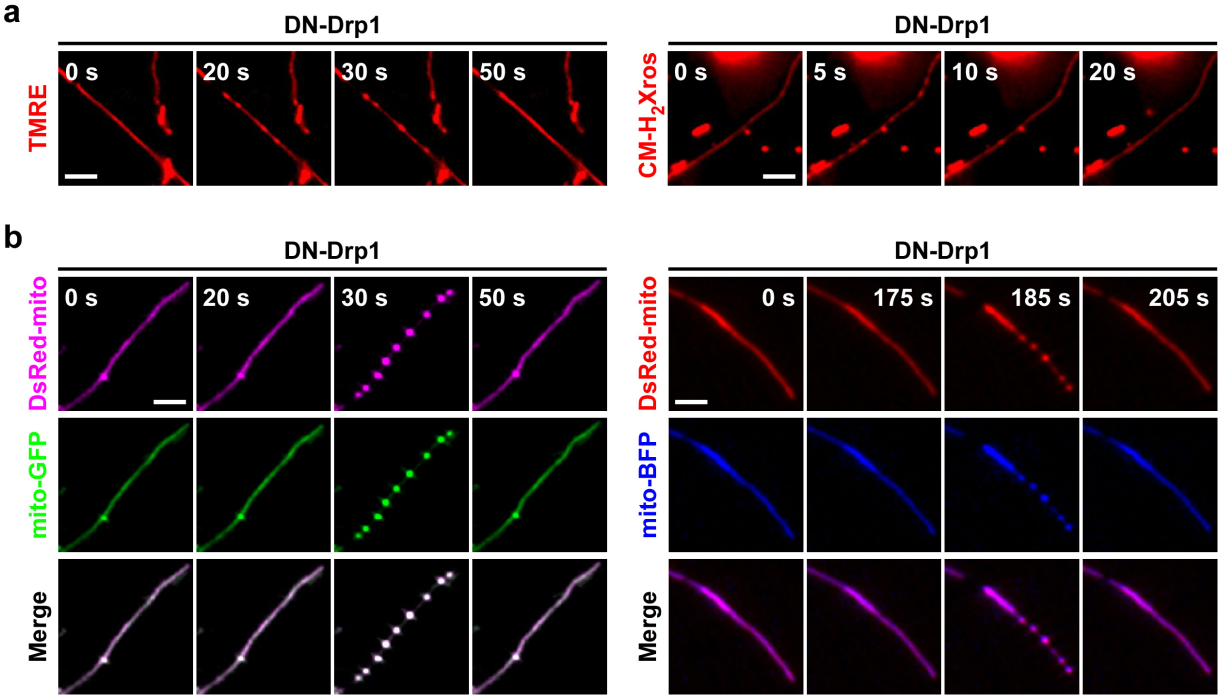

Supplementary Figure 3. CoMIC labelled by chemical and genetic dyes in DN-Drp1-expressing neuron.
(**a**) Time-lapse images of mitochondria labelled by TMRE (left panels) and CM-H_2Xros (right panels) in DN-Drp1-expressing neuron. Scale bars represent 5 μm. (**b**) Time-lapse images of mitochondria labelled by mito-GFP (left panels) and mito-BFP (right panels) with DsRed-mito in DN-Drp1-expressing neuron. Scale bars represent 5 μm.

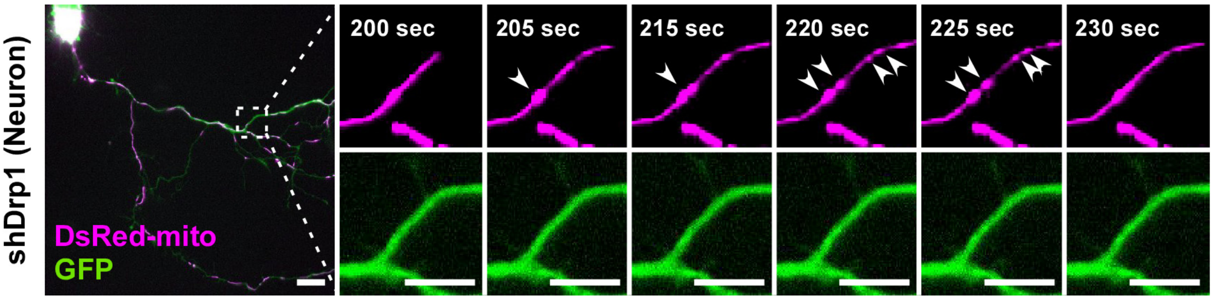

Supplementary Figure 4. CoMIC induction in shDrp1-expressing neuron.

Time-course images of mitochondria in neurons with Drp1-knock down by transfection with small hairpin RNA. Scale bars indicate 10 µm and 5 µm in lower and higher magnification images, respectively.

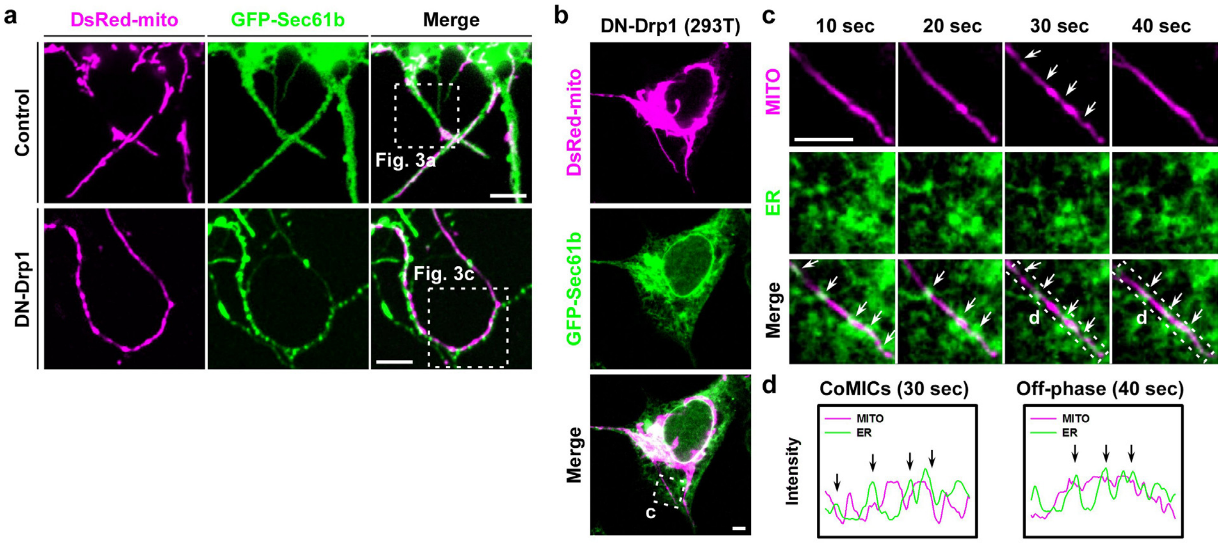

Supplementary Figure 5. Spatial association of CoMIC with ER in 293T cell.

(**a**) Images of mitochondria and ER, which are labeled by DsRed-mito and GFP-Sec61b, in control and DN-Drp1-expressing neuron. Scale bars represent 5 µm. (**b**) Images of mitochondria and ER in DN-Drp1-expressing 293T cell. Scale bars represent 5 µm. (**c**) Time-lapse images of dotted inset of (**b**). Scale bars represent 5 µm. White and yellow arrows indicate the CoMIC spots that are enclosed by ER. (**d**) Intensity profiles of mitochondrial matrix (magenta lines) and ER (green lines) in dotted boxes of (**c**).

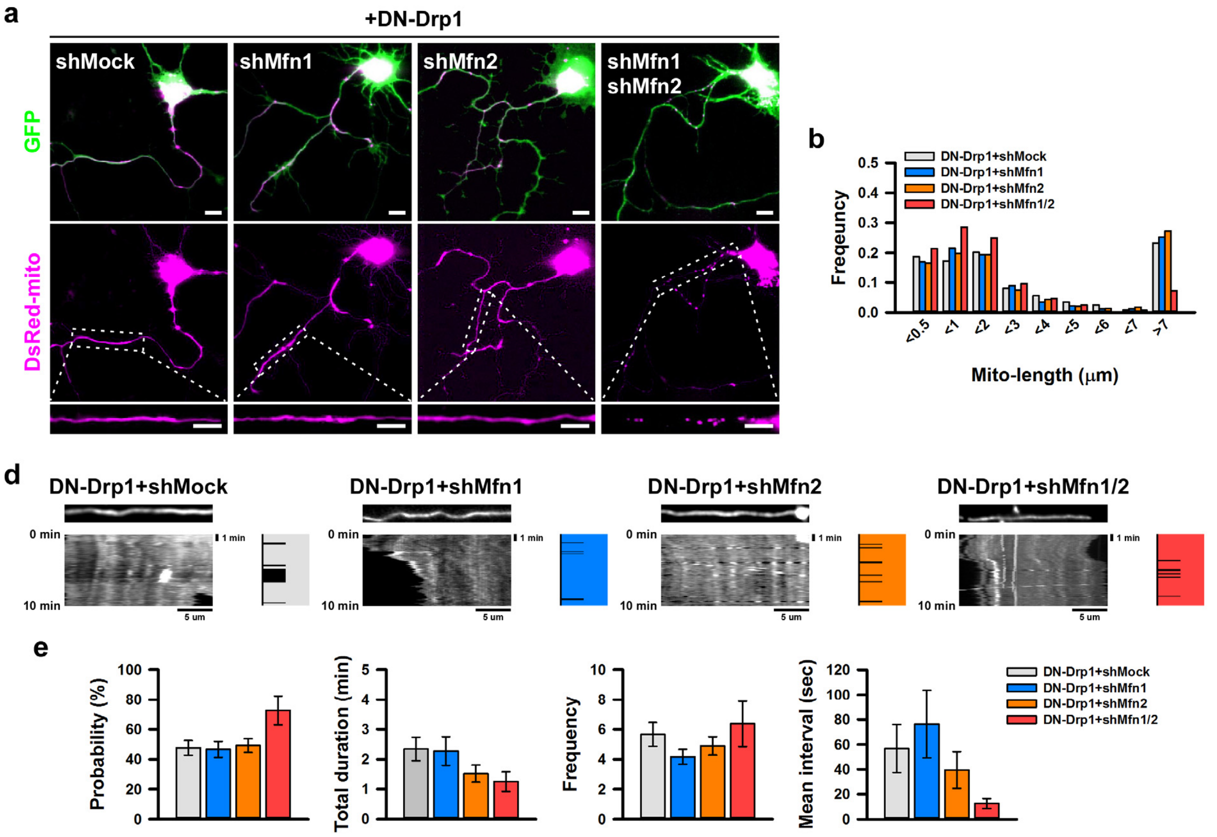

Supplementary Figure 6. Knock-down of Mfn1 and Mfn2 does not affect DN-Drp1-potentiated CoMIC.

(**a**) Protein levels of endogenous Mfn1 and Mfn2 after shRNA expression in neurons (DIV4).

(**b**) Representative morphology of mitochondria in DN-Drp1+shMock-, DN-Drp1+shMfn1-, DN-Drp1+shMfn2-, DN-Drp1+shMfn1/2-expressing neurons. The enlarged panels on the bottom are straightened images of the dotted boxes in the upper panels. All scale bars represent 5 μm. (**c**) Frequency-fractionation analysis of mitochondrial length. (DN-Drp1+shMock, 485 mitochondria; DN-Drp1+shMfn1, 325 mitochondria, DN-Drp1+shMfn2, 393 mitochondria, DN-Drp1+shMfn1/2, 280 mitochondria). (**d**) Representative kymographies of DsRed-mito and patterns of CoMIC (right diagram) in DN-Drp1+shMock-, DN-Drp1+shMfn1-, DN-Drp1+shMfn2- and DN-Drp1+shMfn1/2-expressing neurons. (**e**) Probability of CoMIC in elongated mitochondria from DN-Drp1+shMock- (105 mitochondria), DN-Drp1+shMfn1- (90 mitochondria), DN-Drp1+shMfn2- (122 mitochondria) and DN-Drp1+shMfn1/2-expressing neurons (22 mitochondria), and quantification of the total duration, frequency and average single duration of CoMIC. Error bars indicate standard error.

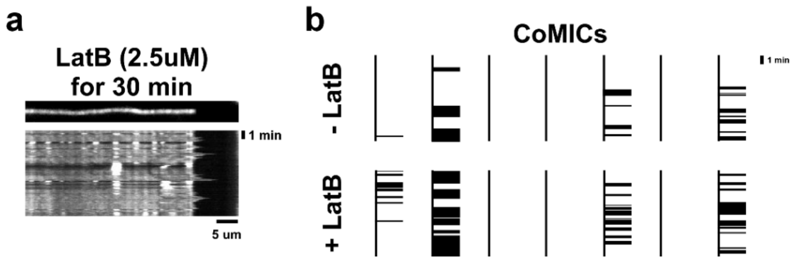

Supplementary Figure 7. Actin filaments are not involved in CoMIC.

(**a**) Morphology and kymography of mitochondria in DN-Drp1-expressing neurons after 30 min pretreatment with 2.5 µM latrunculin B (LatB). (**b**) Patterns of CoMIC before (upper diagrams) and after (lower diagrams) treatment with latrunculin B.

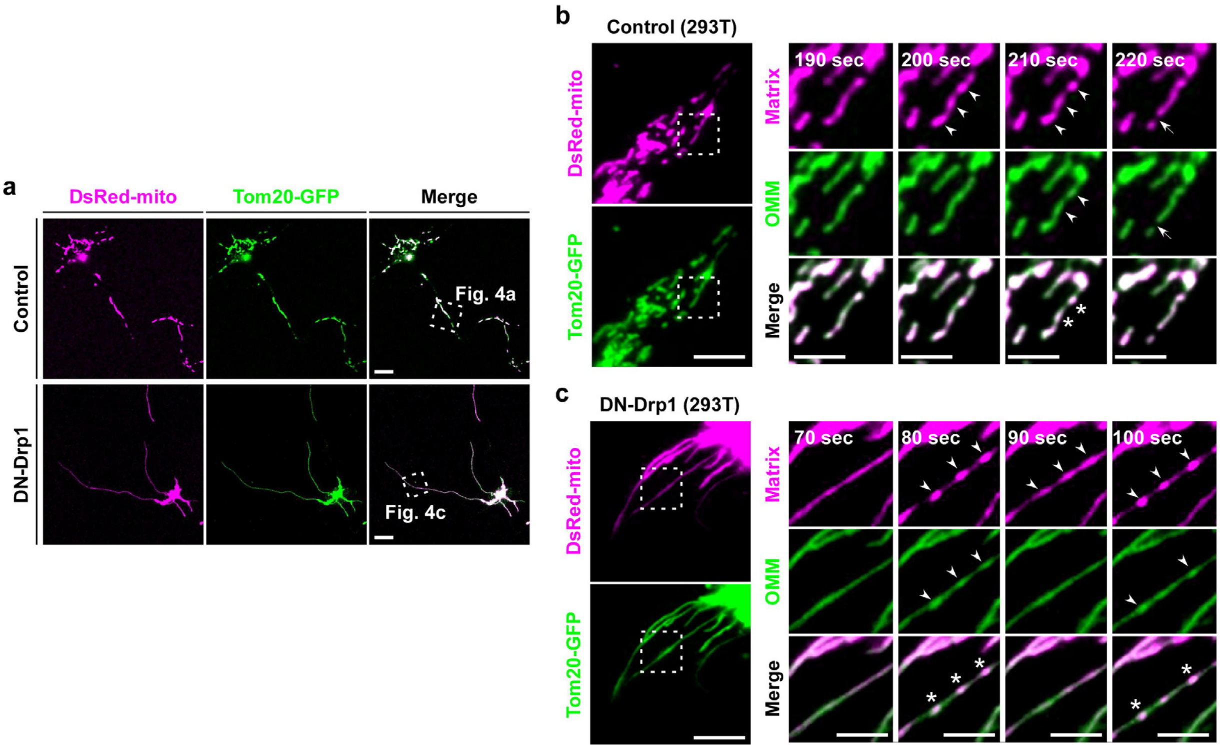

Supplementary Figure 8. Differential constriction on OMM and mitochondrial matrix.

(**a**) Morphology of mitochondrial matrix (DsRed-mito) and OMM (Tom20-GFP) in control and DN-Drp1-expressing neurons. Scale bar represents 10 µm. (**b**,**c**) Morphology of mitochondrial matrix and OMM in control (**b**) and DN-Drp1-expressing 293T cells (**c**). Scale bars represent 10 µm. Enlarged time-course images of mitochondrial matrix and OMM during constriction in the inset of (**b**) and (**c**), respectively. White arrow heads and arrows represent sites of mitochondrial bulging and mitochondrial division. Asterisks indicate synchronized bulging of OMM and matrix. Scale bars represent 5 µm.

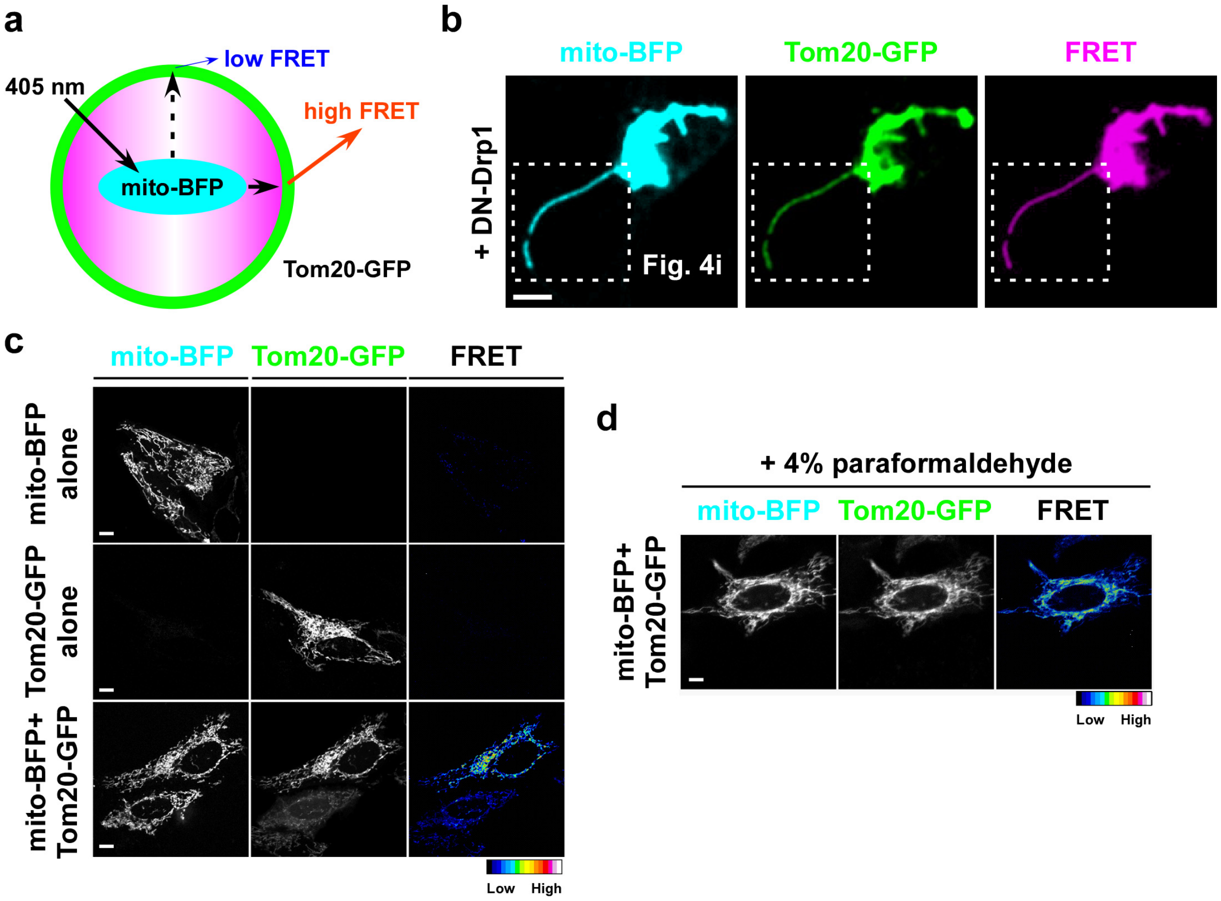

Supplementary Figure 9. FRET-based proximity analysis of OMM and mitochondrial matrix.

(**a**) Schematic diagram of FRET-base proximity analysis of OMM and mitochondrial matrix.

(**b**) Mitochondrial matrix and OMM, labelled by mito-BFP and Tom20-GFP, and their FRET signal (red) in DN-Drp1-expressing neuron. Scale bar represents 5 µm.

(**c**, **d**) Signals of mito-BFP, Tom20-GFP and their FRET in live (**c**) and fixed (**d**) HeLa cells expressing mito-BFP alone, Tom20-GFP alone or mito-BFP with Tom20-GFP. FRET signals are pseudo-colored. FRET signal was detected in only HeLa cells expressing mito-BFP with Tom20-GFP. Scale bars represent 5 µm.

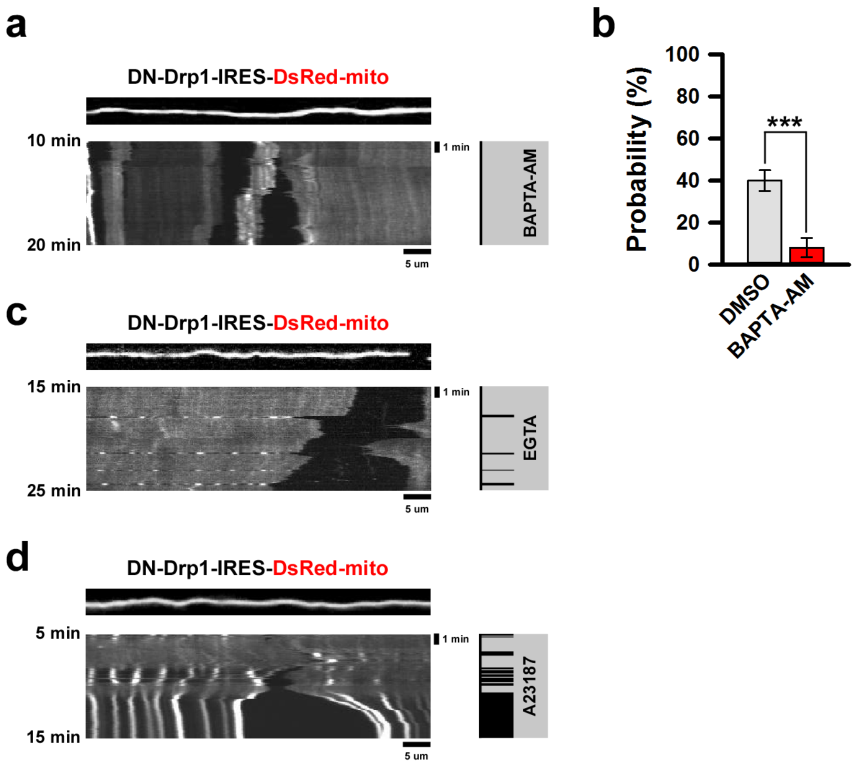

Supplementary Figure 10. Involvement of intracellular Ca^{2+} in CoMIC.

(**a**) Representative kymography of DsRed-mito and pattern of CoMIC (right diagram) in DN-Drp1-expressing neurons under BAPTA-AM (10 μM). (**b**) Probability of CoMIC under no (100 mitochondria) and 1 hour treatment of BAPTA-AM (37 mitochondria). (***p<0.01 by Mann-Whitney Rank Sum Test) Error bars indicate standard error. (**c**,**d**) Representative kymography under EGTA (10 μM) (**c**) and A23187 (1 μM) (**d**).

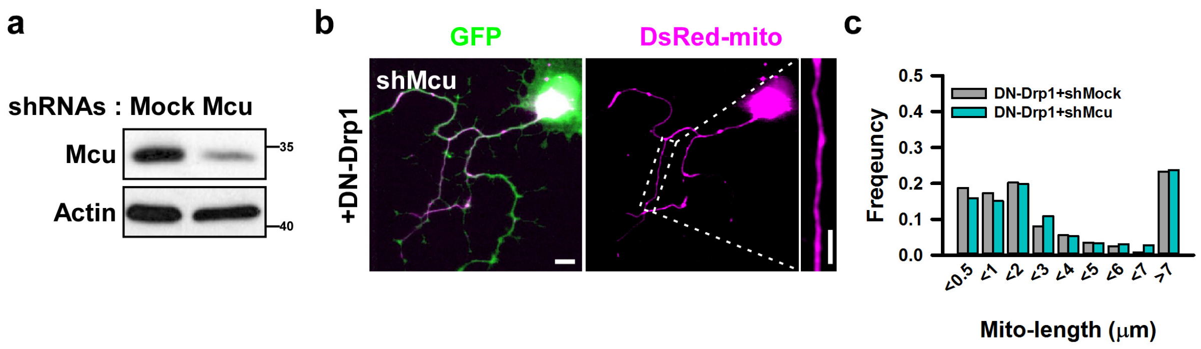

Supplementary Figure 11. Mcu depletion does not affect DN-Drp1-induced mitochondrial hyper-elongation.

(**a**) Protein levels of endogenous Mcu by shRNA expression in neurons (DIV4). (**b**) Representative morphology of mitochondria in DN-Drp1+shMcu-expressing neuron. The enlarged panel on the right are straightened images of the dotted boxes in the left panel. All scale bars represent 5 μm. (**c**) Frequency-fractionation analysis of mitochondrial length. (DN-Drp1+shMcu, 359 mitochondria).

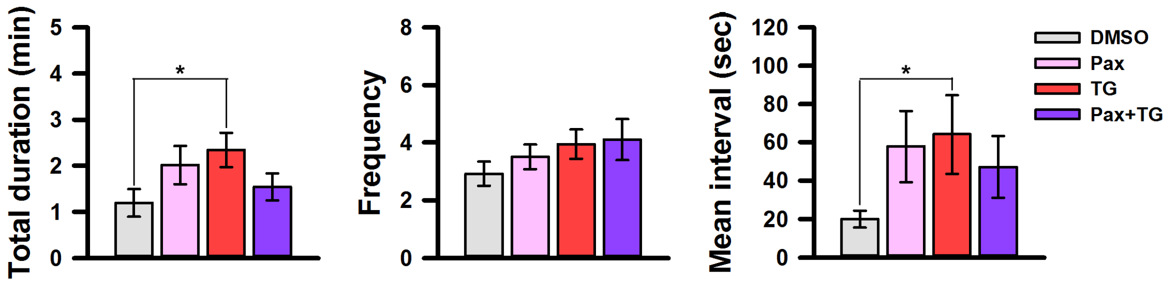

Supplementary Figure 12. Intra-mitochondrial influx of K^+ via mitoBK_{Ca} does not affect potentiation of CoMIC.

Quantification of the total duration, frequency, and average single duration of CoMIC (*p<0.05 by Mann-Whitney rank sum test). Error bars indicate standard error.

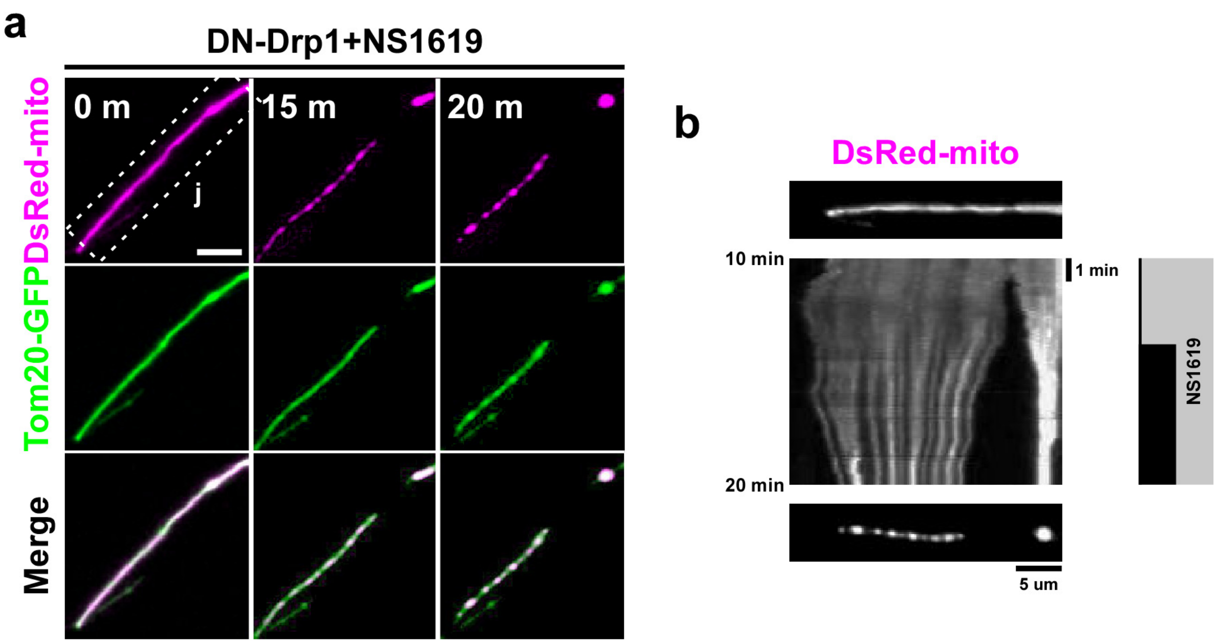

Supplementary figure S13. NS1619 induces CoMIC and mitochondrial fragmentation.

(**a**) Time-lapse images of DsRed-mito and Tom20-GFP. (**b**) Kymography of DsRed-mito in DN-Drp1-expressing neurons under long exposure of NS1619 (100 µM). Scale bars represent 5 µm.

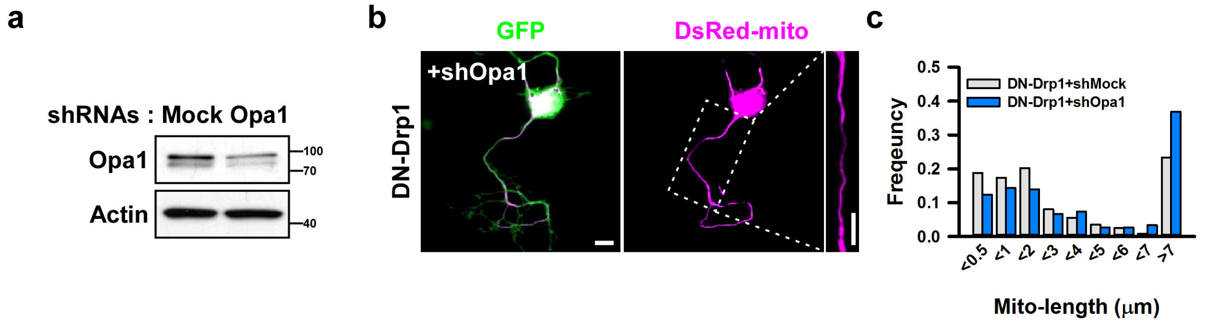

Supplementary Figure 14. Opa1 depletion does not affect DN-Drp1-induced mitochondrial hyper-elongation. (**a**) Protein levels of endogenous Opa1 by shRNA expression in neurons (DIV4). (**b**) Representative morphology of mitochondria in DN-Drp1+shOpa1-expressing neuron. The enlarged panel on the right are straightened images of the dotted boxes in the left panel. All scale bars represent 5 μm. (**c**) Frequency-fractionation analysis of mitochondrial length. (DN-Drp1+shOpa1, 301 mitochondria).

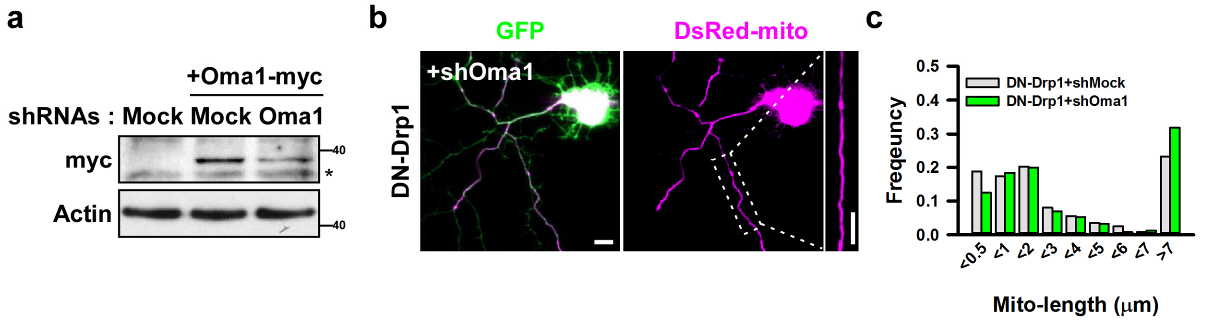

Supplementary Figure 15. Oma1 depletion does not affect DN-Drp1-induced mitochondrial hyper-elongation. (**a**) Protein levels of ectopic myc-tagged rat Oma1 by shRNA co-expression in 293T cell. Asterisk indicates non-specific band. (**b**) Representative morphology of mitochondria in DN-Drp1+shOma1-expressing neuron. The enlarged panel on the right are straightened images of the dotted boxes in the left panel. All scale bars represent 5 μm. (**c**) Frequency-fractionation analysis of mitochondrial length. (DN-Drp1+shOma1, 402 mitochondria).

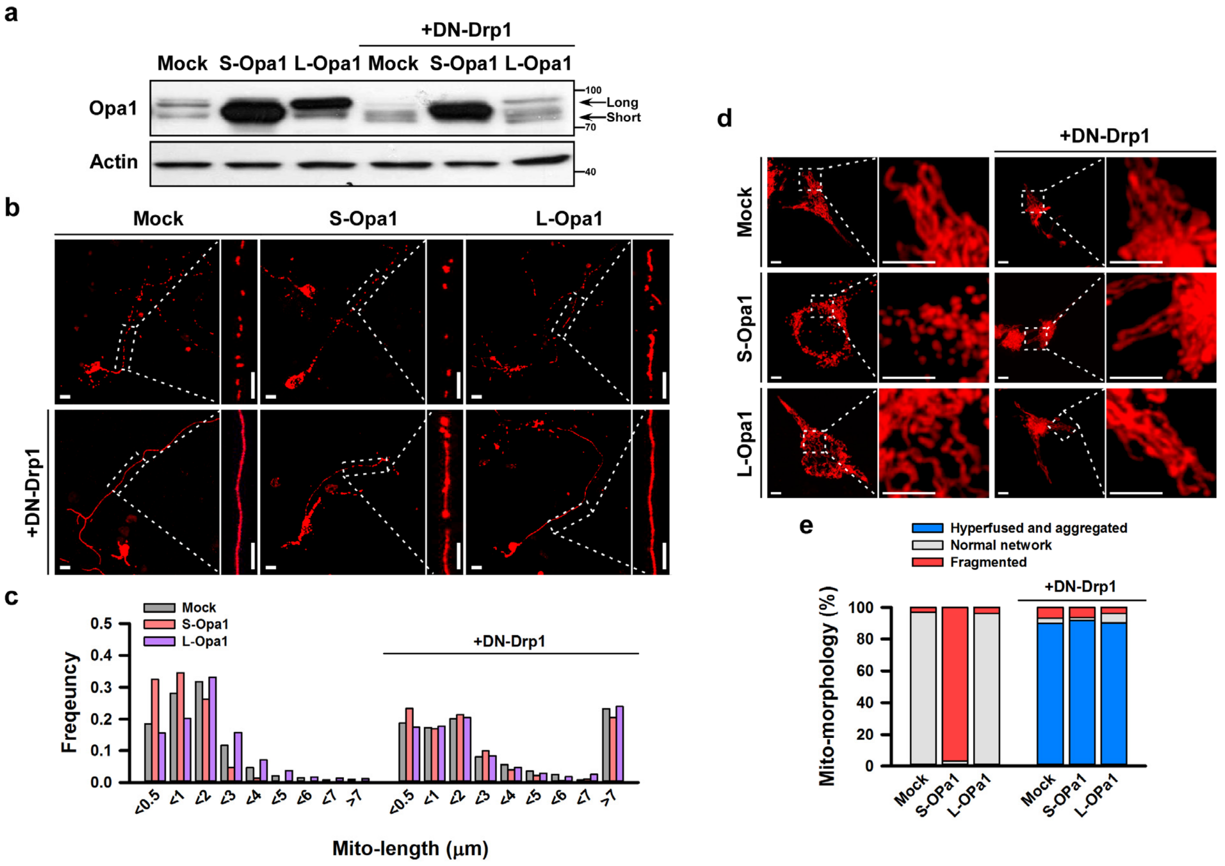

Supplementary Figure 16. S-Opa1 induces Drp1-dependent mitochondrial fragmentation in 293T cells. (**a**) Western blot of Opa1 in control, S-Opa1- and L-Opa1-transfected 293T cells without or with DN-Drp1. (**b**,**d**) Mitochondrial morphologies in control, L-Opa1-, S-Opa1, DN-Drp1-, DN-Drp1+S-Opa1- and DN-Drp1+L-Opa1-expressing neurons (**b**) and 293T cells (**d**).The enlarged panels on the bottom are from images of the dotted boxes in the upper panels. All scale bars represent 5 μm. (**c**) Frequency-fractionation analysis of mitochondrial length. (Mock, 1,051 mitochondria; S-Opa1, 1,048 mitochondria; L-Opa1, 1,026 mitochondria; DN-Drp1, 485 mitochondria; DN-Drp1+S-Opa1, 556 mitochondria; Drp1+L-Opa1, 555 mitochondria). (**e**) Quantification of cells exhibiting hyperfused, normal and fragmented mitochondria in control, L-Opa1-, S-Opa1, DN-Drp1-, DN-Drp1+S-Opa1- and DN-Drp1+L-Opa1-transfected 293T cells.

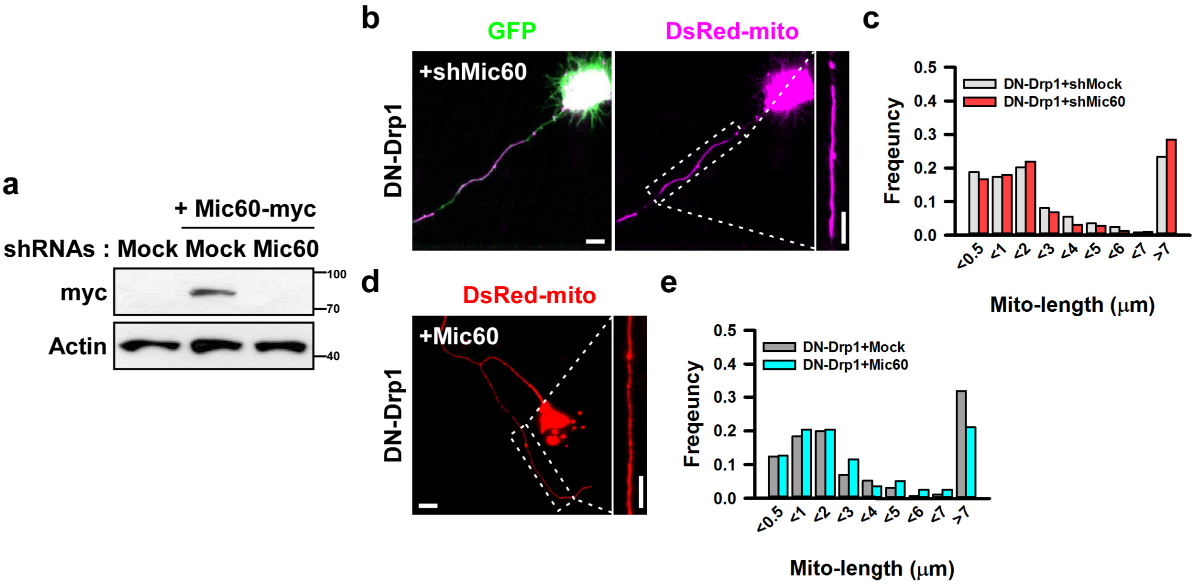

Supplementary Figure 17. Mic60 perturbation does not affect DN-Drp1-induced mitochondrial hyper-elongation.

(**a**) Protein levels of ectopic myc-tagged rat Mic60 by shRNA co-expression in 293T cell. (**b**,**d**) Representative morphology of mitochondria in DN-Drp1+shMic60- (**b**), DN-Drp1+Mic60-expressing neuron (**d**). The enlarged panel on the right are straightened images of the dotted boxes in the left panel. All scale bars represent 5 μm. (**c**,**e**) Frequency-fractionation analysis of mitochondrial length. (DN-Drp1+shMic60, 323 mitochondria; DN-Drp1+Mock, 402 mitochondria; DN-Drp1+Mic60, 388 mitochondria).

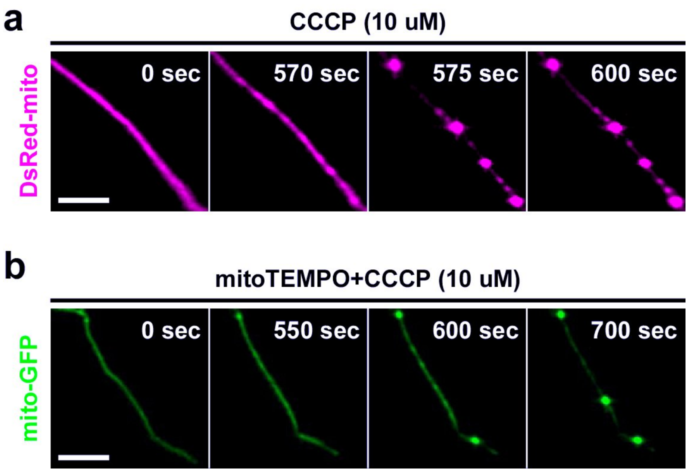

Supplementary Figure 18. CCCP induces CoMIC and mitochondrial fragmentation independently mitochondrial ROS.

(**a**,**b**) Time-lapse images of mitochondria in DN-Drp1-expressing neuron treated by CCCP alone (**a**) and CCCP with mitoTEMPO (**b**). All scale bars represent 5 μm.
